# Transcriptome sequencing for high throughput SNP development and genetic mapping in Pea

**DOI:** 10.1186/1471-2164-15-126

**Published:** 2014-02-12

**Authors:** Jorge Duarte, Nathalie Rivière, Alain Baranger, Grégoire Aubert, Judith Burstin, Laurent Cornet, Clément Lavaud, Isabelle Lejeune-Hénaut, Jean-Pierre Martinant, Jean-Philippe Pichon, Marie-Laure Pilet-Nayel, Gilles Boutet

**Affiliations:** 1 Biogemma, route d’Ennezat, CS 90126, Chappes 63720, France; 2 INRA UMR 1349 IGEPP, BP35327, Le Rheu Cedex 35653, France; 3 Limagrain Europe, centre de recherche route d’Ennezat, CS 3911, Chappes 63720, France; 4 INRA UMR 1347 Agroécologie, Bat. Mendel, 17 rue Sully BP 86510, Dijon 21065, France; 5 INRA, UMR 1281 SADV, Estrées-Mons BP 50136, Péronne 80203, France

**Keywords:** *Pisum sativum*, *Medicago truncatula*, Next generation sequencing, Genetic diversity, Composite genetic map, Synteny, Marker assisted selection

## Abstract

**Background:**

Pea has a complex genome of 4.3 Gb for which only limited genomic resources are available to date. Although SNP markers are now highly valuable for research and modern breeding, only a few are described and used in pea for genetic diversity and linkage analysis.

**Results:**

We developed a large resource by cDNA sequencing of 8 genotypes representative of modern breeding material using the Roche 454 technology, combining both long reads (400 bp) and high coverage (3.8 million reads, reaching a total of 1,369 megabases). Sequencing data were assembled and generated a 68 K unigene set, from which 41 K were annotated from their best blast hit against the model species *Medicago truncatula*. Annotated contigs showed an even distribution along *M. truncatula* pseudochromosomes, suggesting a good representation of the pea genome. 10 K pea contigs were found to be polymorphic among the genetic material surveyed, corresponding to 35 K SNPs.

We validated a subset of 1538 SNPs through the GoldenGate assay, proving their ability to structure a diversity panel of breeding germplasm. Among them, 1340 were genetically mapped and used to build a new consensus map comprising a total of 2070 markers. Based on blast analysis, we could establish 1252 bridges between our pea consensus map and the pseudochromosomes of *M. truncatula,* which provides new insight on synteny between the two species.

**Conclusions:**

Our approach created significant new resources in pea, i.e. the most comprehensive genetic map to date tightly linked to the model species *M. truncatula* and a large SNP resource for both academic research and breeding.

## Background

Molecular markers are widely used in plant research for candidate gene or QTL identification through linkage or association mapping as well as analysis of population structure and evolution. It has also become a major resource for accelerated plant breeding through marker assisted selection [1]. SNPs (Single Nucleotide Polymorphism) are now the genetic markers of choice since they are a virtually unlimited, evenly distributed along the genome, bi-allelic and co-dominant resource. Moreover, an increasing number of technologies are now available for fast and inexpensive genotyping, from medium (Veracode, 384 SNP) to very high throughput (i-Select Illumina, Axiom Affymetrix). Until recently, massive SNP discovery was limited to a few species for which a reference genome was available, such as maize [2,3] or Arabidopsis [4]; http://naturalvariation.org/hapmap). Tremendous advances in next generation sequencing technologies now make it feasible to sequence even complex genomes at a reasonable cost [5]. In addition, the challenge due to large genomes with very high levels of repeated sequences has led to the development of different approaches to reduce genome complexity. Methyl-filtration which targets hypo-methylated gene-enriched regions was applied to develop markers in maize [6,7] or switchgrass [8]. cDNA sequencing appears as a simple way to address the expressed genic fraction. Transcriptome sequencing was intensively described in a wide range of species, including models (Arabidopsis, rice, tomato), and crops with large genomes [9-14], including field pea [15,16]. A number of these sequencing studies have led to the development of SNP markers with applications in diversity panel structuration or genetic mapping in cereals [17-19], oilcrops [20,21], and model [22] or cultivated legumes [23-27]. Although considered an important legume crop, there has been a surprisingly low effort yet in developing SNP markers for field pea mapping or diversity studies [28].

*Pisum sativum* is the third grain legume crop in the world after soybean and common bean and is a major source of proteins for both human food and livestock feed. Moreover, pea is particularly relevant in cropping systems due to its capacity to fix nitrogen through symbiosis. Nevertheless, the species suffers from significant yield instability due to high susceptibility to biotic and abiotic stresses [29-35]. Resistance QTLs have been described, but with still large confidence intervals due to low resolution of existing genetic maps. It remains a challenge both (i) to understand underlying mechanisms and identify the candidate genes involved, and (ii) to reduce QTLs confidence interval sizes and develop breeding programs using powerful molecular markers.

Field pea can be considered to be an orphan species considering its limited genomic resources. Its genome covers 4.3 Gb, which is around 10 times larger than the genome of the model species *M. truncatula*[36], including repeats mostly based on transposon-based sequences [37]. To date no full genome sequence and only poor EST resources (18,576 EST sequences in Genbank in June 2013) are available. Recent reports show that large new sequencing resources are under development [15,16,37] and that a consortium for pea genome sequencing is being built (http://www.coolseasonfoodlegume.org/pea_genome). However, these efforts have not yet reached the development of large numbers of new molecular markers to saturate pea maps and improve QTL mapping both towards research and breeding objectives. Available genetic maps in pea remain low to medium density, and are based mainly on a few hundred SSRs [38] and SNPs [28,39]. It is therefore strategic for field pea breeding to develop large new resources for mapping and genetic improvement.

Analyzing polymorphism within this species through a whole genome resequencing strategy is difficult and genome complexity reduction is mandatory. Franssen [16] first described large scale transcriptome sequencing with the objective to provide a comprehensive reference set for further analysis in the species. Kaur [15] further investigated marker development through transcriptome sequencing of different tissues from four field pea cultivars and identified 2397 gene-related SSR markers, 96 of which were genotyped, with 50 eventually displaying polymorphism within a set of six genotypes. Even though the two studies gave rise to a significant enrichment in EST contigs and resources, they did not provide a large marker resource.

Our objective, to complement the existing resources and to better fit with research and breeding demand for markers, was to develop a comprehensive SNP database in pea with extended validation in breeding and genetic mapping positions. For this purpose, we deeply sequenced eight genotypes representing the genetic diversity present in modern breeding material, and developed a dedicated bioinformatics pipeline for assembly and SNP identification.

## Results

### Discovery of 35,455 highly reliable SNP

Eight *P. sativum* genotypes were selected for sequencing, in order to address genetic diversity present in European breeding material, including six spring sown, one winter sown field pea as well as one fodder pea cultivar. cDNA was normalized prior to the sequencing step in order to smooth out differences between highly and poorly expressed genes. The normalization efficiency was assessed by Q-PCR on 48 genes selected for showing a wide range of expression levels (Additional file 1: Figure S1). Low Cp values (highly expressed genes) increased from 10–15 to 15–20 between control and normalized cDNAs for all genotypes, a shift of five PCR cycles corresponding approximately to a 30 fold decrease in abundance. At the same time no significant change was observed for high Cp values (poorly expressed genes), suggesting that cDNA normalization did not remove rare transcripts and therefore raised their overall relative abundance.

The eight normalized cDNA samples, one for each cultivar, were subjected to 454 sequencing and data assembly. From half a sequencing run dedicated to each sample, we generated 365,255 to 591,513 raw reads per sample, reaching a total of 1,369 Mb from 3,826,797 reads. Median read length per genotype ranged from 361 to 420 bp and 68% to 78% of the read lengths were between 300 and 600 bp depending on the sample. After data cleaning for small/long reads, PCR duplicates and low complexity sequences, we kept 78% of available sequences. The last cleaning steps consisted in masking repeated sequences and removing chloroplast derived sequences: 1,068 Mb of high quality sequences were eventually used for *de novo* assembly (Table 1).

**Table 1:** Statistics on raw and pre-processed sequencing data across the eight samples

	**Raw data**				**Pre-processed data**				**Processing details**				
	**Nb reads**	**Nb bases**	**Average length**	**% 300-600 bp**	**Nbreads**	**Nbbases**	**Average length**	**% 300-600 bp**	**% of bases removed**	**Pyrocleaner**	**Medicago repeats**	**PCR oligos**	**Chloroplast**
Champagne	496 034	181 943 498	366.8	74.75%	414 114	150 011 369	362.2	73.23%	17.56%	10.85%	1.21%	5.25%	0.88%
Cherokee	574 074	197 526 312	344.1	70.70%	458 682	155 095 699	338.1	67.38%	21.48%	15.00%	1.11%	5.31%	0.91%
Hardy	526 038	187 918 202	357.2	73.75%	443 607	155 739 374	351.1	70.89%	17.14%	10.39%	1.33%	5.24%	0.80%
Kayanne	413 098	139 462 293	337.6	68.18%	343 271	114 332 181	333.1	65.09%	18.04%	10.73%	1.36%	5.95%	0.72%
Lumina	474 380	168 623 198	355.5	70.91%	376 824	132 088 228	350.5	68.87%	21.66%	14.04%	1.30%	6.02%	1.25%
Panache	591 513	205 278 821	347.0	70.78%	453 509	153 535 495	338.6	66.84%	25.22%	18.01%	1.32%	5.68%	1.32%
Pocket	365 255	138 664 207	379.6	77.54%	267 463	98 969 773	370.0	75.17%	27.88%	20.31%	1.73%	6.29%	0.94%
Terese	386 405	149 269 177	386.3	78.52%	284 948	107 897 288	378.7	77.00%	27.71%	19.74%	1.6%	5.71%	1.81%
**Total**	**3 826 797**	**1 368 685 708**	**359.3**	**72.85%**	**3 042 418**	**1 067 669 407**	**352.8**	**70.12%**	**21.12%**	**14.76%**	**1.36%**	**5.64%**	**1.07%**

Eighty percent of the data could be assembled (2,466,808 reads) in 68,850 contigs, representing a cumulated length of 58 Mb. N50 contig size was 956 bp, average size was 842 bp, and the longest one reached 5,250 bp (Additional file 2: Figure S2). Overlap between genotypes was high as 70% of contigs were covered by reads from at least four different genotypes (Additional file 3: Figure S3).

Out of the 68,850 contigs, hits were found for 54,156 (78.7%) against UNIPROT and 50,636 (73.5%) against *M. truncatula* predicted proteins with e-value lower than 1e-5. Informative description was assigned to 40,135 contigs (Additional file 4: Table S1). 36,094 contigs were annotated from UniProt (hits below 1e-25) and 4,041 contigs from *M. truncatula* proteins. Altogether, 16,966 annotations were “similar to” and 23,169 “highly similar to” (see Methods). A total of 14,613 non-redundant matches against *M. truncatula* proteins were found, which is slightly more than the 10,594 [16] and 11,737 [15] found on previous assemblies of the pea transcriptome.

### SNP calling

A total of 74,861 putative SNPs were called, among which 35,455 met the selection criteria for robustness. These 35,455 highly reliable SNPs were found in 10,522 contigs, among which 9,813 (95%) had a hit below 1e-15 against UNIPROT (Plants only) and were further annotated using Blast2GO: 7,338 (71%) could be annotated with a GO term (default settings) (Additional file 5: Figure S4). The coordinates of the 10,522 contigs’ best homologs along the *M. truncatula* chromosomes are described in the supplementary data (Additional file 6: Table S2). A majority of the detected polymorphisms (58%) had a minor allele frequency of 1/8, which means they were brought by only one genotype. Almost half of them were brought by the fodder pea Champagne, the most distant genetically to the other seven field pea genotypes. Eleven thousand eight hundred and three SNPs were polymorphic between Champagne and Terese, which could be used for further genetic map densification purposes using the Champagne x Terese derived RIL population.

### Selection and validation of a 1920 SNP set in a GoldenGate assay

**Figure 1:**
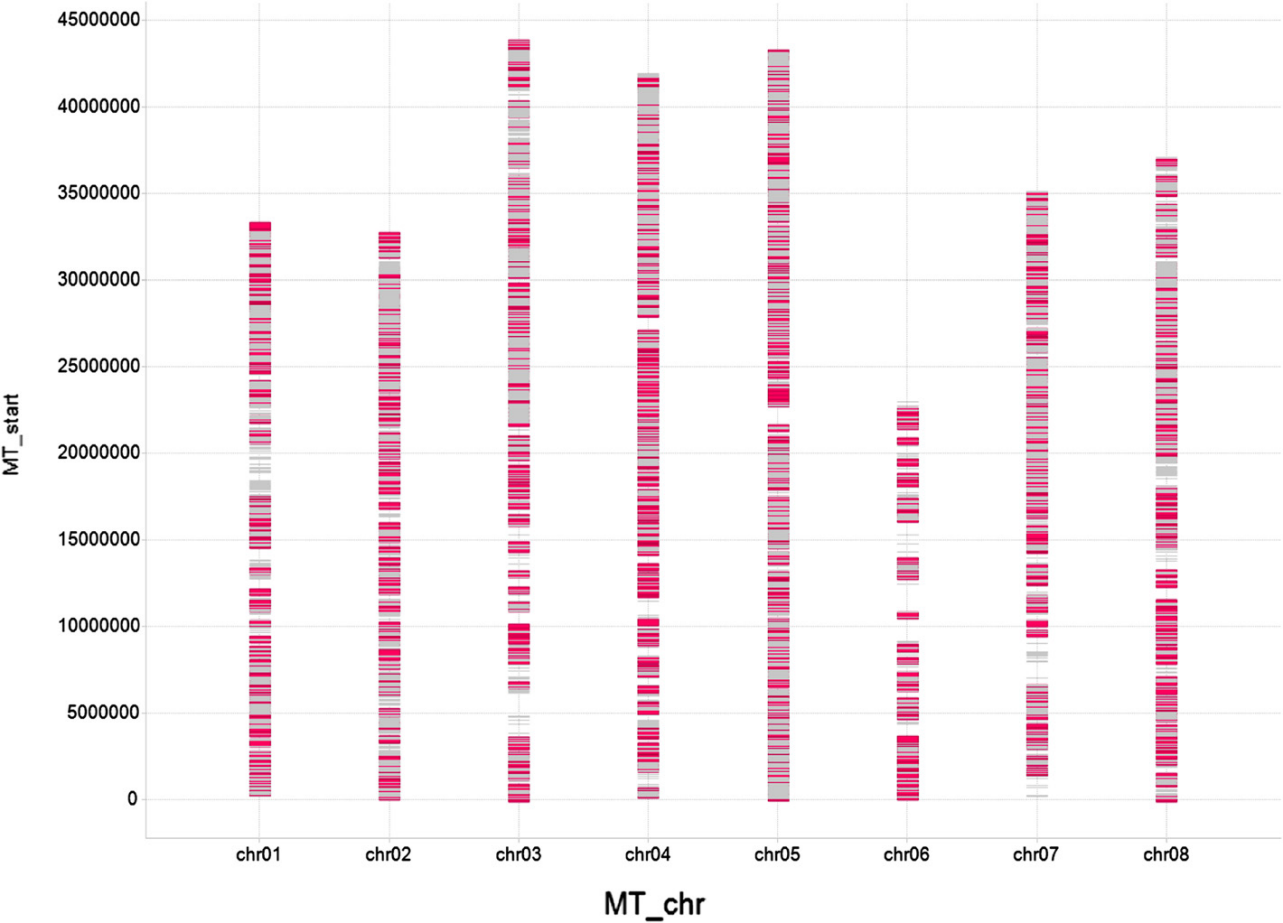
**Conserved distribution along *M. truncatula *** **pseudo-chromosomes (MT_chr) of the 10,522 pea polymorphic cDNA contigs (grey bars) and the 1,920 pea cDNA contigs (pink bars) selected for genotyping.**

Based on both technical and biological criteria (see Methods) we selected 1920 SNP, out of the 35 k, for genotyping. In order to ensure representativeness of this SNP subset, we looked at GO term assignment as well as at the distribution along *M. truncatula* chromosomes. GO terms distributions were generally conserved between the complete set of 7,338 annotated contigs and the genotyped subset of 1920 contigs for each type of annotation (Additional file 5: Figure S4). Only 3 terms (transporter activity, plasma membrane, and growth) were over-represented and two terms (thylakoid and DNA binding) under-represented in the genotyped subset (Fisher’s exact test p-values ranging from 4.4e-2 to 2.2e-2, Additional file 7: Table S3). In addition, we found little difference in the distribution of pea contig homologs along *M. truncatula* chromosomes between the two data sets, contigs from both data sets showing even distributions along the chromosomes (Figure 1) except for a few *M. truncatula* chromosomal regions that remained uncovered both by the whole dataset and by the 1920 SNP subset, the largest on chromosomes 1, 3, 6 and 7. The 1920 subset therefore constituted an unbiased sampling of the whole SNP resource generated.

From 1920 SNPs selected for the GoldenGate assay, 1620 (84.5%) were successfully genotyped (Additional file 8: Table S4) on either a diversity panel of pea accessions, or on one or more of four pea RIL populations. The remaining 300 SNPs (15.5%) failed due to missing or non-interpretable signal. Genotyped SNPs were classified between A and H quality levels (Additional file 9: Table S5), most of them (1250) having the highest quality (A). Only 59 SNPs presented a Major Allele Frequency above 0.95 and 50 markers proved to be monomorphic which indicates a low false positive rate during our process of SNP calling and selection. Among the 1620 successfully genotyped SNP markers, 1538 revealed the expected biallelic codominant polymorphism in the pea diversity panel, and 1360 showed polymorphism in at least one of the four parental pairs of RIL populations. Few markers presented genotyping abnormalities (Additional file 8: Table S4): 50 could not be genotyped in one out of the four RIL populations; 86 presented a dispersed cloud of data for one allele and should be used with caution (among which 45 were classified in C quality level); 63 corresponded to multilocus or copy number variations; 55 showed a dominant (presence/absence) allele for at least one RIL population, among which 51 were classified in “D” quality level.

### Validation of a 1920 SNP set in a GoldenGate assay for pea genetic diversity assessment, and selection of an informative 297 SNP sub-set

**Figure 2:**
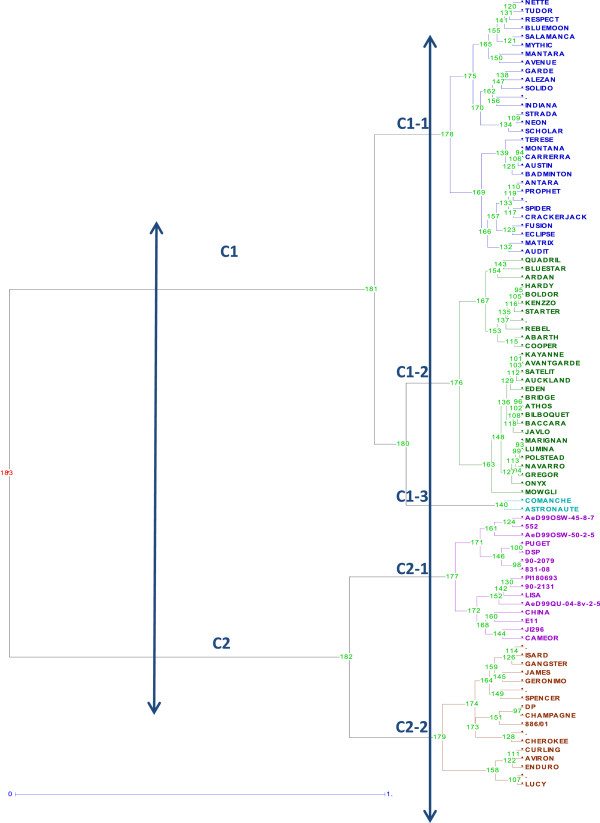
**Classification of a diversity panel of 92 pea accessions using 1,538 SNPs.** Rogers’ distances were computed for all pairs of accessions and a Ward hierarchical classification procedure was used to classify the accessions in clusters (Cx) and subclusters (Cx-x). Unnamed branches are non-registered breeding lines currently in the registration process.

Classification of the 92 accessions of the diversity panel through a Ward hierarchical clustering showed the ability of the 1538 genotyped SNPs to group pea genotypes into two main clusters (Figure 2): Cluster 1 consisted of 60 accessions including 56 spring sown field pea cultivars and could be divided into three sub clusters: Subcluster 1–1 contained 29 accessions of spring sown field pea cultivars from various breeding companies and unexpectedly one winter sown pea accession (Indiana). Most of these cultivars were developed for and registered in Northern Europe (UK, Denmark, Holland, Germany) and used for various ends including human food and exports. Subcluster 1–2 consisted of 28 accessions of spring sown field pea cultivars representing different breeding companies’ programmes and end-uses, including feed peas that are registered and developed in France. Subcluster 1–3 consisted of one winter sown field pea accession (Comanche) and one spring sown field pea (Astronaute) that seem to be genetically close. Cluster 2 grouped 32 accessions and could be divided into two sub clusters: Subcluster 2–1 consisted of 15 accessions of garden pea accessions, spring field pea breeding and recombinant lines from a breeding program aiming at incorporating *Aphanomyces euteiches* resistance from garden pea resistance sources. Cluster 2–2 consisted of 15 winter sown field pea cultivars or breeding lines, together with two fodder pea accessions (DP, Champagne).

The factorial analysis confirmed the same global structuration as the Ward hierarchical clustering, with axis 1 and 2 explaining 22.3% and 6.5% of variance respectively (Additional file 10: Figure S5). A first group (G1) clearly identified spring field pea cultivars belonging exclusively to Cluster 1. Cultivars belonging to Subclusters 1–1 and 1–2 were still separated within G1 except for cv Ardan. A second group (G2) clearly grouped winter field pea cultivars and fodder peas consistent with Subcluster 2–2 of the hierarchical clustering. A third group (G3) comprised garden and field pea accessions from the *A.euteiches* resistance breeding program consistently with Subcluster 2–1 of the hierarchical clustering. Interestingly, the factorial analysis identified intermediate positions of the two cultivars belonging to Subcluster 1-3 (Astronaute, Comanche) between the G1 and the G2, and confirmed the unexpected position of the winter sown cultivar Indiana (intermediate between the 3 groups). Finally, seven accessions, mostly lying in Subcluster 1-1, and mainly corresponding to marrowfat pea cultivars, showed intermediate positions between G1 and G3.

A classification of 1538 genotyped SNPs was performed using a transposed matrix through a Ward hierarchical clustering using the genotyping data of the 92 accessions of the diversity panel. Forty-eight sub-clusters of SNPs were defined (Additional file 8: Table S4), within which two to ten SNPs were chosen based on SNP quality level, and position on the consensus map to select a 297 SNP subset. This defined 297 SNP subset (Additional file 8: Table S4) classified the 92 pea accessions of the diversity panel as well as the 1538 SNP set into the same five clusters (Additional file 11: Figure S6). Fifty-nine accessions were conserved identically on the Maximum Agreement sub-Tree (data not shown) resulting in the comparison of the two Ward hierarchical clustering. From the 92 accessions, only three moved from one sub-cluster to another (Additional file 11: Figure S6): Ardan and Rebel from sub-cluster 1–2 to sub-cluster 1–1 (these two cultivars belong to the G1-1 group corresponding to SC1-1 in the factorial analysis) (Additional file 2: Figure S2), and E11 from SC2-1 to SC2-2 (this accession is positioned between G2 and G3, corresponding to SC2-2 and SC2-1 in the factorial analysis) (Additional file 10: Figure S5).

### Validation of the 1920 SNP set in a GoldenGate assay for genetic mapping in pea

**Figure 3:**
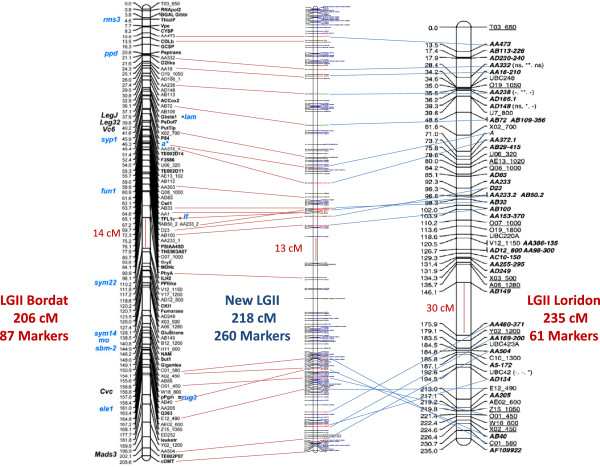
**Colinearity of common markers between our study (middle) and the Bordat *et al. *** **(**[39]**; left) and Loridon *et al. *** **(**[38]**; right) composite maps on LGII.**

A new high-density composite pea genetic map, covering 1255 cM and including the newly developed SNP markers was constructed from a matrix composed of 2464 markers × 360 genotypes from four RIL populations. For each genotyped SNP, the flanking sequence, the best blast hit on the *M. truncatula* genome of its associated contig, the corresponding annotation, the quality index, the rate of polymorphism and its position on the new *P. sativum* composite map are described (Additional file 8: Table S4). The percentages of SNPs that showed segregation distortion (P < 0.01) were estimated at 14.8%, 6.8%, 5.5% and 4.5% in populations derived from the crosses JI296xDP, ChampagnexTerese, ChinaxCameor and BaccaraxPI180693, respectively. A total of 2070 markers could be reliably mapped including 1340 SNP from the present study (65%) and 730 previously mapped markers (Additional file 12: Figure S7), giving a density of 1.65 markers per cM. This map presented only one gap larger than 10 cM between two contiguous markers, and only 12 gaps larger than 10 cM between contiguous newly developed SNPs (Table 2). Marker density was high and similar for all *P. sativum* Linkage Groups (PsLGs), ranging from 1.6 to 2.1 markers/cM (1.1 to 1.3 for the developed SNP) with the noticeable exception of PsLGII for which the marker density was 1.2 (0.7 for developed SNPs). Positions of the 730 previously mapped markers on our consensus map were generally collinear with their published positions [32,33,38] [Mohamadi *et al.* A composite genetic map in pea including new eSSR loci., *in preparation*]: 14 to 25 markers mapped on each Linkage Group were common with both the Loridon *et al.*[38] and Bordat *et al.*[39] consensus maps (Additional file 13: Figure S10). Except for a few local inversions, collinearity of these markers was maintained along the three maps, with the notable exception of the PsLGII for which a block inversion was observed at the distal part LGII with the Loridon *et al.*[38] consensus map but not with the Bordat *et al.*[39] consensus map (Figure 3). Map sizes were similar between the present consensus map (1255 cM), the Loridon *et al.* map (1430 cM) [38] and the Bordat *et al.* map (1389 cM) [39] but the number of mapped markers was increased 4-fold in comparison to those previous composite reference maps, respectively comprising 462 [38] and 536 [39] markers.

**Table 2:** Number of markers and newly developed SNP, map length, distribution of markers and SNPs per linkage group and on the whole genome map

	**LG1**	**LG2**	**LG3**	**LG4**	**LG5**	**LG6**	**LG7**	**Whole**
**Number of markers**	235	260	339	270	265	298	404	2071
**Number of developped SNPs**	161	150	214	180	198	166	270	1340
**Length (cM)**	147	218	203	169	156	142	220	1255
**Number of Markers/cM**	1.6	1.2	1.7	1.6	1.7	2.1	1.8	1.7
**Number of gaps > 10 cM between two contiguous Markers**	0	1	0	0	0	0	0	1
**Number of developed SNPs/cM**	1.1	0.7	1.1	1.1	1.3	1.2	1.2	1.1
**Number of gaps > 10 cM between two contiguous developed SNPs**	0	6	3	2	0	0	1	12

### Synteny between a 1252 SNP-based pea genetic map and the *M. truncatula* physical map

From the 1340 mapped SNP, 1252 provided a link between their original cDNA contigs on the pea Linkage Groups and the position of their best blast hits on the *M. truncatula* pseudo-chromosomes. Over 75% of these 1252 links presented a highly conserved organization between the seven Pea LGs, and the eight *M. truncatula* pseudo-chromosomes (Additional file 14: Figure S8). This was summarized by a dotplot of macrosyntenic relationships (Additional file 15: Figure S9). Clear blocks of synteny were observed with varying levels of rearrangements: PsLGI, PsLGII, PsLGIV, PsLGV, and PsLGVII corresponded to *M. truncatula* chromosomes Mtrchr5, Mtrchr1, Mtrchr8, Mtrchr7, and Mtrchr4 respectively, with some local inversions. A number of rearrangements could be observed, such as those between PsLGIII compared to Mtrchr2 and Mtrchr3 as well as PsLGVI compared to Mtrchr6 and Mtrchr 2. The Mtchr3 in *M. truncatula* corresponded to the major part of the pea PsLGIII but showed many breaks and reversals blocks. The central part of PsLGVI corresponded to the entire Mtchr6, its upper part to the upper portion of the Mtchr2, and its lower part to the central part of Mtchr2. Finally the lower and middle portions of Mtchr2 corresponded to PsLGVI, and its upper part to the upper part of PsLGIII, with two collinear blocks framing two reversed blocks (Additional file 16: Figure S11).

## Discussion and conclusions

Sequencing of eight cDNA normalized libraries from genotypes representative of modern pea breeding material allowed the assembly of a large collection of cDNA contigs, and identification of over 35,000 reliable SNP markers. A subset of SNPs were genotyped with the Golden Gate assay to generate a high density composite genetic map including 1340 newly developed SNPs and anchored on the *M. truncatula* physical map.

### Normalized cDNA sequencing: an appropriate strategy for development of markers in an orphan species

Transcriptome sequencing is an efficient strategy for genome reduction in non-model species since it focuses on coding regions rather than on the entire genome. This is especially true in plants where the size and the repetitive nature of the genomes reduce the coding fraction. In the case of pea, 75 to 97% of the genome [40,41] is covered by repeats. The drawback of cDNA sequencing is that the number of sequences by gene reflects its expression level in the extracted tissue. cDNA normalization is an efficient way of limiting over-representation of genes with high expression rates, and ensuring a representation of genes with low expression rates. Indeed, the comparison of pea cDNA sequencing with and without normalization showed a loss of 30% of represented genes when no normalization was made [16]. In our study, the QPCR performed on 48 genes representative of a wide range of expression levels clearly shows that normalization reduced the abundance of genes with high expression rates without affecting the abundance of moderately and weakly expressed genes. Our results also indicate that there is still a significant correlation between raw and normalized data, as shown by Franssen *et al.*[16].

### Data assembly and SNP calling

Since at the time this project was initiated very little sequence data was available for pea (5,004 nucleotide entries and 18,552 EST sequences in genbank in 2010), the chosen strategy was de novo sequencing and assembly. Like for many other non-model plants transcriptome sequencing projects [42], we chose to use the Roche/454 platform with the GS-FLX Titanium chemistry which provides long read lengths (400 bp in average) which is critical for de novo assembly. While many different assembly strategies combining different tools have been tested in previous transcriptome studies [42], we chose to use the MIRA assembler, which was present in the top three assemblers used in recent 454 transcriptome projects [43] and has proven to work well on pea [16] and on other complex species like rapeseed [20] or wheat [44].

Previous pea transcriptome assemblies using the Roche 454 technology reported an average contig length of 324 bp from 250 bp read length [16], or an average contig length of 719 bp [15]. We obtained a longer average contig length (842 bp) and N50 (956 bp), closer to this last report. Furthermore, a comparison of our assembly to previous ones showed that it covers them well with 70,337 contigs (86%) out of 81,449 from Franssen *et al*. [16] and 12,776 (95%) out of 13,445 from Kaur *et al*. [15] having a hit against our assembly (megablast with e-value lower than 1e-5 and option -p 95). Reciprocally from 68,850 contigs from this study, 49,235 (71%) and 39,868 (58%) had a hit against Franssen *et al*. [16] and against Kaur *et al*. [15] assemblies respectively. The nearly 20,000 contigs from our assembly that show no similarity with previous datasets may be due to a higher sequencing effort in this study (3.8 M reads compared to 720 K reads in Kaur *et al*. [15]). We can anticipate that new sequencing technologies, such as the Illumina Miseq that now generates 2×300 bp at a lower cost will help to design new sequencing projects with both long reads and deep coverage.

The objectives of the earlier studies mentioned above aimed at SSR development and validation [15], or exhaustive representation of expressed genes [16], whereas this study clearly focused its experimental design and bioinformatics analysis on identification of SNP markers easy to genotype with high throughput technologies. Although they all contributed to generating pea cDNA contig sequences, it appears that these three recent studies could be complementary in many points to better characterize the pea transcriptome.

We also developed a dedicated script for SNP calling adapted to the data we generated. For validation purpose, we assessed by genotyping 1920 SNP (5% of the overall SNP resource) using Illumina GoldenGate VeraCode technology on a large number of pea accessions, from which 1620 were confirmed as true SNP. This high validation rate (84.5%), although expected on a diploid species with such a technology, fully validated the chosen bioinformatics pipeline for SNP calling.

### High density genetic map bridged to *M. truncatula* by synteny

This study presents the first high-density pea composite map mainly based on SNPs likely to enable large-scale studies by both academic and breeder users.

The map size obtained was similar to the ones observed in previous reference composite maps based on SSR [38] or genic markers [39], but with a 4-fold increase in marker density, raising overall resolution to 1 cM. This new high density composite map also makes a significant step forward following the founder mapping of reduced sets of SNP markers in pea by Deulvot *et al.*[28] and Legrand *et al.*[45]. The presence of a RIL population as well as more than a hundred markers in common with previous composite maps greatly facilitated the comparison and potential use of the newly developed SNP in a range of pea populations. A high level of collinearity was observed for the 730 markers that were common to other *P. sativum* maps [32,38,39], which make the newly developed 1340 mapped SNPs a useful tool for future studies focusing on a genomic region or trait in pea. This new composite map will allow resolution of previous or future conflicting data in pea mapping. For instance the block inversion shown in the distal part of the PsLGII on the Loridon *et al.*[38] map is probably a mis-assembly of two blocks distant by more than 30 cM due to a lack of markers on this map, whereas this gap was filled and the inversion resolved in our study.

One thousand two hundred and fifty-two SNPs derived from pea cDNAs were anchored to the genome of the model species *M. truncatula* which opens the door to large-scale syntenic studies. Previous studies reported a high level of macrosynteny between Pea and Medicago genomes [36,39,46,47]. The most comprehensive study to date [39] placed 5460 pea unigenes on the *M. truncatula* physical map but only 149 bridges between the genomes were truly mapped in pea. Since genotyped SNPs were selected on the basis of pea contigs’ homology to *M. truncatula*, the number of potential bridges between the two species increases now to approximately 10,000. Since collinearity for some loci has not always been found, a reciprocal blast from Medicago to pea should be performed, as described by Bordat *et al*. [39], to confirm our results. In any case, the high density of collinear bridges generated here will allow further investigation of apparently complex genome reorganization spots between the two species, such as for instance the complex structuration of PsLGIII and PsLGVI.

### Classification of modern pea cultivars and breeding lines

We addressed modern field pea breeding genetic diversity by genotyping 92 genotypes. The panel was structured into clusters, separating cultivated types of spring field peas, winter field peas, garden peas and lines of interest for *A. euteiches* resistance. Only two apparent classification mismatches were detected (Comanche and Indiana), probably due to registration as winter pea of cultivars that are derived from the spring pea gene pool [Declerck P: pers.com.]. Subclustering within spring pea cultivars did not separate gene pools from different breeding companies or according to geography, which shows that the narrow gene pool used in spring pea breeding in France is shared by main pea breeders. Subclustering within winter pea cultivars did not separate gene pools from different breeders either, but clearly showed that winter pea cultivars are mostly derived from fodder peas. Finally, the main division lies between spring sown and winter sown pea breeding, although one may feed the other for some crosses which may be the origin of the few mismatches observed. This structuration into cultivated types is consistent with a number of previous reports regarding the classification of a large diversity of germplasm [48-50] or focusing mainly on the classification of cultivars [51], using different kinds of PCR based molecular markers. Newly developed SNPs therefore show efficiency in structuring diversity in pea cultivars, even using the proposed reduced set of 297 informative SNPs.

### A comprehensive resource for academic research and breeding in pea

This study generated three major resources that will address both research issues regarding genetic control of traits of interest, and breeding issues for the introgression and management of these traits into cultivated gene pools.

First, the new composite genetic map, that reaches the cM level resolution, will undoubtedly have a major impact on genetic analysis of traits in pea to fine map and refine QTL confidence intervals, and to identify underlying candidate genes. Moreover, almost all the 35 K SNPs identified can be ordered on the Medicago genome according to blast results and can therefore be a reservoir of SNPs for marker densification within regions of interest. This newly available resource of bridge markers between species will allow synteny based QTL mapping, candidate gene identification and cloning between pea and *M. truncatula* in regions of interest, such as those identified for *A. euteiches* resistance [33,52], or for frost resistance [53]. It will also allow breeders to select new markers from that reservoir which will better describe their introgressions and improve marker-assisted selection.

Second, the 68 K pea cDNA contigs generated constitute an additional and complementary sequence resource to the recently published ones [15,16], which will help for the definition of the pea gene space. A potential use of this resource could for instance be a targeted genotyping of a RIL population through resequencing for high density genetic mapping. Very high density genetic maps appear mandatory for scaffold anchoring in sequencing projects and the emerging pea genome sequencing project (http://www.coolseasonfoodlegume.org/pea_genome) will benefit from it. Indeed, the increased reliability and density of the map developed, combined with syntenic projections within the newly sequenced model species *M. truncatula,* will help in defining the structure of the pea genome, and to investigate in more details complex reorganizations like fracture zones and inverted blocks between the two genomes*.* Chromosomal rearrangements within pea lines will also be investigated by comparing the consensus map to individual maps [54] of different RIL populations.

Third, different SNP sets were generated: 35,000 technically reliable, 10,000 anchored to the *M. truncatula* physical map, 1,350 mapped on the pea genetic map, 1,538 polymorphic across a collection of modern pea cultivars, 297 optimally representing differentiation between these cultivars. These data sets could be used by pea breeders for a variety of applications, such as selection of genetically distant lines, follow up of haplotypes in the progenies, or monitoring of the presence of favorable alleles for agronomic traits for variety registration purposes.

The combined use of these three resources provides a powerful tool for Marker Assisted Selection. It gives comprehensive knowledge for the selection of subsets of SNP markers to use from polymorphism, mapping and hierarchical information. Finally, the proposed resources will undoubtedly help in directing the creation of new pea ideotypes cumulating alleles at new QTLs for traits of interest, adapted to various climates and cropping systems, with stabilized and high yields.

## Methods

### Plant material and tissue collection for sequencing

Six spring sown (Lumina, Hardy, Panache, Rocket, Kayanne and Terese), one winter-sown (Cherokee) and one fodder (Champagne) pea cultivars were selected for sequencing. The Champagne genotype was incorporated as a parent of the Champagne x Terese mapping population (allowing further genetic mapping) and potential resistance source to frost and ascochyta blight disease. The eight *P. sativum* genotypes were grown in a growth chamber (photoperiod 16 h light/day, 15°C night, 20°C day, hygrometry 60% min) and at least five plants per genotype were collected 15 days after sowing. Tissues were flash frozen in liquid nitrogen and stored at −80°C until further use.

### Two sets of plants used for genotyping

The first set consisted of four Recombinant Inbred Line (RIL) mapping populations developed by Single Seed Descent from crosses between various parental lines: 91 RILs from the cross ‘JI296’ x ‘DP’ [29]; 91 RILs from the cross ‘Champagne’ x ‘Terese’ [38]; 91 RILs from the cross ‘China (JI1491)’ x ‘Cameor’ [28]; 91 RILs from the cross ‘PI180693’ x ‘Baccara’ [32]. The population Champagne x Terese has already been used for the establishment of previous composite maps [38,46].

The second genotyping sample set was composed of a diversity panel of 72 modern pea cultivars, and of 20 parental genotypes of mapping populations and recombinant inbred and breeding lines of interest for resistance to *A. euteiches* (Additional file 17: Table S6).

### RNA extraction

Total RNA was extracted from tissue powder with the RNeasy plant kit (Qiagen) according to the manufacturer’s instructions. RNA purity and integrity were checked by capillary electrophoresis on a BioAnalyzer (Agilent). RNA concentration was determined on a Nanodrop® spectrometer and OD260/OD280 ratio calculated for purity assessment.

### cDNA normalization

cDNA normalization was performed from total RNAs with MINT and TRIMMER kits from Evrogen according to the manufacturer’s instruction, except that the number of PCR cycles for material amplification was adapted to our material. First, full length double stranded (ds) cDNA were synthetized from 2 μg of total RNA using the MINT kit [55]. First strand was synthetized from a fusion primer containing an oligo (dT) stretch to anneal RNA polyA tails. A poly (dC) stretch was incorporated at the end of the first strand, and used for priming the synthesis of the second strand. Full length (ds) cDNA were subsequently amplified by PCR, purified on Qiaquick columns (Qiagen) and checked for quality and yield before normalization. Normalization was done with the TRIMMER kit (Evrogen) which is based on DSN technology [56]. The method involves denaturation-reassociation of cDNA, Duplex Specific Nuclease (DSN) degradation of the ds-fraction corresponding to abundant transcripts and PCR amplification of the single strand (ss) DNA fraction. We started from 600 ng (ds) cDNA for normalization and after denaturation, incubated samples at 68°C for five hours for renaturation. After degradation of (ds) complexes by DSN, we made two runs of PCR amplification for optimal recovery. Normalized cDNA was then purified on Qiaquick columns (Qiagen) and yield was measured by spectrophotometry.

### Evaluation of normalization efficiency

We verified the efficiency of normalization by measuring gene representation on a set of genes covering a large range of expression levels by Q-PCR on native and normalized samples. Forty-eight genes analyzed by Q-PCR in previous studies (unpublished data) were considered. Two μl of cDNA from both conditions (native and normalized) were used for Q-PCR using Fast Start Universal SYBR green Master mix (Roche), in a 10 μl reaction.

### Library preparation and sequencing

Sequencing library preparation was performed using Roche 454 GS-FLX kits according to the manufacturer’s recommendations. For each cultivar, we started with 1 μg (ds) cDNA that was submitted to fragmentation using a nebulization method (Roche). An average size of 700 pb was obtained for each sample, as verified by capillar electrophoresis (Agilent Bioanalyzer). Libraries were sequenced on a 454 GS-FLX sequencer (Roche) with the Titanium chemistry (400 bp read length). Each cultivar was sequenced on half a PicoTiterPlate (PTP). A total of four PTP, each generating in average 400 Mb sequences (1 million reads, 400 pb length), was necessary to sequence the eight cultivars. Raw data were produced as sff files.

### Sequence cleaning

Raw data were first processed through different cleaning steps. Pyrocleaner v1.0 [57] was used to remove sequences with a length outside a given range (mean read length +/− 2 × standard deviation), as well as potential PCR duplicates, and low complexity sequences. RepeatMasker v3.2.9 [58] was used to identify and mask known repeats using the Medicago repeat library from TIGR Plant Repeats (ftp://ftp.plantbiology.msu.edu/pub/data/TIGR_Plant_Repeats/TIGR_Medicago_Repeats.v2). SmartScreener [59] and SeqClean [60] (http://compbio.dfci.harvard.edu/tgi/software/) were used in order to remove remaining PCR oligos introduced during the cDNA normalization protocol. Finally Seqclean was also used to screen sequences for chloroplast contamination using the Pea chloroplast genome sequence (NCBI RefSeq NC_014057.1).

### Sequence assembly

These sequences were then assembled using MIRA [61] in “est” mode. The eight genotypes were assembled altogether. This strategy has the advantage of keeping track of all reads and base calls in alignments and will facilitate high quality SNP discovery later on. Again, in order to obtain high quality alignments, we used very stringent constraints on sequence assembly. MIRA provides a very wide range of parameters which are by default set according to each sequencing technology, but which can also be tuned differently to take into account genome specificities. Due to high polymorphism in Pea, different versions of MIRA with different settings were tested (data not shown). Version 3.4rc3 seemed to produce the best result. The command line used was: *mira -project = peapol -job = denovo,normal,est,454 --notraceinfo -GE:not = 10 -SB:lsd = yes 454_SETTINGS -AL:mrs = 90:mo = 30 -AS:mrpc = 4*. The option *mrs* stands for *minimum relative score*, and describes the minimum percentage of matching between two reads to be considered for assembly. It was set to 90 (80 by default). The option *mo*, which stands for *minimum overlap*, was increased to 30 (20 by default). These two options increased the stringency in sequence alignment. They also reduced the computational time required to complete the assembly as well as the amount of memory used. The last option *mrpc*, which stands for *minimum reads per contig*, was set to 4 (2 by default) to generate a comprehensive SNP resource only from contigs with at least four reads.

### Sequence annotation/homology search against *M. truncatula*

Despite the fact that our aim here was to discover SNPs and not to build a representative unigene set of pea transcriptome, we conducted a functional annotation of contigs mainly to check assembly quality. Contigs were compared to UNIPROT (plant only) and Medicago genome protein predictions (release 3), using blastx and a minimum e-value of 1e-5. Only informative description was given to contigs with the prefix ‘similar to’ when it had a hit with an e-value between 1e-25 and 1e-50, and with the prefix ‘highly similar to’ for hits with an e-value lower than 1e-50. Annotations were considered informative when they did not contain one of these keywords: unknown, anonymous, hypothetical, bac, cosmid, predicted, unnamed, uncharacterized.

### SNP discovery

To our knowledge, at the time the data was produced and analyzed, although different tools were being developed for SNP discovery, none of them were specifically designed or well established for calling SNPs from 454 data on homozygous diploid crop lines. Therefore SNP discovery was conducted using a custom perl script which we have used with success on other projects in diploid and polyploid crop cultivars [20,44,62]. This script can directly process MIRA’s assembly ACE output format by going through each contig alignment, looking for variant positions and then filtering these positions according to default thresholds and/or user-controlled parameters: minimum base quality, NQS (Neighbor Quality Standard) and coverage criteria. Here, we set the parameters to use a “20/15 NQS criterion” for a 11-base window as initially described by [63] in order to define high quality bases. Then, since we had good quality, long (400 bp) sequences, we set the minimum high quality bases depth to 2x per genotype. This means that the script filtered out all variant positions that did not have at least 2 genotypes, each with 2 different base calls with a minimum phred score of 20 and within good quality context (5 bases on each side with a minimum phred score of 15). By applying these filters a first set of putative SNPs was defined. Since we used fixed lines, a second filter was applied to keep only positions for which each accession was strictly homozygous independently of phred score. Finally, due to the high error rate of 454 sequencing on homopolymers, all indels were excluded from the final selection defined as *robust SNPs*. Further annotation was done on contigs containing at least one robust SNP using Blast2GO [64].

### Selection of a 1920 SNP set and validation in a GoldenGate assay

A set of 1920 SNPs was chosen to design five custom VeraCode assays for the Illumina BeadXpress Reader. The objective for SNP selection was to get an even distribution of markers all along the genome, based on synteny with the model species *M. truncatula*. Final SNP selection was based on 1) designability on Illumina technology, 2) elimination of redundancy on the basis of blast hits of pea contigs against *M. truncatula* proteins, 3) one single SNP per contig, 4) removal of SNPs present in the close vicinity of exon-exon junctions. This last criterion was added considering further genotyping assays are performed on genomic DNA. At the end, considering that among the eight genotypes included in the analysis Champagne was known as genetically distant from the others, SNPs with the minor allele coming exclusively from Champagne were removed from the selection.

### DNA extraction and genotyping

DNA was extracted from leaf tissue using a CTAB method as described by Rogers and Bendich [65]. DNA was quantified with the Quant-iT™ PicoGreen® Assay (Invitrogen, Carlsbad, USA), using the Appliskan multiplate reader (Thermo Scientific, Courtaboeuf, France). DNA concentrations were adjusted to 50 ng/μL for each sample. For each assay, five plates of 96 samples containing 50 μL of genomic DNA normalized to 50 ng/μL were provided for genotyping using the “GoldenGate Genotyping Assay for VeraCode Manual Protocol” (Illumina Inc., San Diego, USA) [66]. The automatic allele calling for each locus was accomplished using the Genome Studio software (Illumina Inc., San Diego, USA). The homozygous and heterozygous clusters were checked visually and they were manually edited when necessary. Technical replicates and signal intensities were verified; only the most reliable calls were retained. A quality mark was then given to each SNP as follows: (A) Excellent genotyping; (B) Polymorphism detected but low fluorescence; (C) Polymorphism detected but low cluster separation; (D) Polymorphism detected but some accessions (> 10%) were not genotyped; and (E) Failed or No polymorphism detected (Additional file 9: Table S5).

### Composite genetic map construction

Markers that were used from different published [33,38,39,46,47,67-70] or unpublished [Mohamadi *et al.*, *in preparation*] maps and 1360 from our 1920 SNP markers were added to constitute a combined genotyping matrix for the four Recombinant Inbred Line (RIL) mapping populations. The 1/1 segregation ratio of each marker within each population was checked using a Chi-square test (P > 0.01 and P > 0.001). Genetic linkage analyses were performed using the “group” commands of CarthaGene software [71], with a minimum LOD score threshold of 3.0 and a recombination frequency < 0.3. Marker order was refined using the “annealing 100 100 0.1 0.9” command of CarthaGene software. The Kosambi function was used to calculate centiMorgan (cM) distances between markers. MapChart 2.2 was used to draw the composite map [72].

### Statistical analyses

A statistical approach was used to describe the relationship between accessions. Marker polymorphism information content (PIC) was calculated with Powermarker V3.25 [73]. In order to get a representation of the genetic structure of the 92 pea accession collection (Additional file 4: Table S1), an analysis based on the 1538 newly generated polymorphic SNPs was performed with DARwin5 software [74]. The dissimilarity matrix generated using the Rogers-Tanimoto method with 10,000 bootstraps was used for factorial analysis and for construction of the Ward hierarchical clustering tree [75]. The same software and parameters were used using a transposed matrix to structure the 1538 SNP set with the 92 pea accessions (data not shown).

### Availability of supporting data

This Transcriptome Shotgun Assembly project has been deposited at DDBJ/EMBL/GenBank under the accession GAMJ00000000. The version described in this paper is the first version, GAMJ01000000. The raw data was deposited at SRA under accessions [SRR934439-SRR934446].

## Competing interests

The authors declare that they have no competing interests.

## Authors’ contributions

JD carried out all the bioinformatics analysis and co-wrote the manuscript. NRI conceived and coordinated the study, carried out the statistical analysis of the normalization, co-wrote the manuscript. AB co-wrote the manuscript. GA, JB, ILH and MLPN participated to the production of the genotyping material. LC carried out all the molecular biology experiments. JPP coordinated the molecular biology experiments. CL participated in the construction of the composite genetic map. JPMA coordinated the genotyping experiments and helped to draft the manuscript. GB coordinated the study, carried out all the genetic and statistical analyses, and co-wrote the manuscript. All authors read and approved the final manuscript.

## Supplementary Material

Additional file 1: Figure S1 Expression levels of 48 genes between initial (before normalization, X axis) and normalized (Y axis) conditions for the two genotypes Champagne (blue) and Cherokee (red). Expression level was assessed by Q-PCR and estimated by Cp (Crossing point), where high Cp indicates a low expression level, and low Cp a high expression level. Click here for file

Additional file 2: Figure S2 Length distribution of the 68,850 contigs resulting from the de novo assembly of 454 sequencing data from 8 pea genotypes. Click here for file

Additional file 3: Figure S3 Distribution of the pea genotypes’ contribution to the 68,850 contigs. Click here for file

Additional file 4: Table S1 Informative description given to 40,135 contigs. Click here for file

Additional file 5: Figure S4 GO term distribution comparison between the 7,338 annotated contigs set (from the 10,522 contigs containing robust SNPs, orange bars, only terms present in more than 1% of contigs shown) and the 1,920 subset that was genotyped (green bars). Click here for file

Additional file 6: Table S2 Original contig, 201 bp context sequence, and best blast hit annotation from the *M. truncatula* genome for the 35,544 robust SNPs called from 10,522 pea contigs. Click here for file

Additional file 7: Table S3 Fisher’s Exact Test with Multiple Testing Correction of FDR (Benjamini and Hochberg). Significantly differentially represented terms from comparing a test group (1920 contigs subset) to a reference group (10,522 contigs set) for Gene Ontology terms enrichment. Click here for file

Additional file 8: Table S4 Original contig, 201 bp context sequence, genotyping quality values, mapping position on the pea consensus map and classification in 48 groups based on genotyping data of the 1920 genotyped SNP markers. Click here for file

Additional file 9: Table S5 SNP Quality criteria. Click here for file

Additional file 10: Figure S5 Groupings across the 92 accessions and cultivars diversity panel revealed by a Factorial Analysis on genotyping data from 1538 SNP markers. Click here for file

Additional file 11: Figure S6 Classification of a diversity panel of 92 pea accessions using 297 SNPs. Rogers’ distances were computed for all pairs of accessions and a Ward hierarchical classification procedure was used to classify the accessions in clusters (Cx) and subclusters (Cx-x). Click here for file

Additional file 12: Figure S7 *P. sativum* composite map presenting 1340 newly developed SNP markers (shown in blue). Most markers shown in red are SSR markers common with a previous consensus map (Loridon *et al.*[38]). Distances are in cM (Haldane). Click here for file

Additional file 13: Figure S10 Collinear positions between the *P.sativum* composite genetic map and *M. truncatula* physical map. For pea linkage groups, 1u = 1 cM; for *M. truncatula* pseudo-chromosomes, 1u = 0.1 Mb. Click here for file

Additional file 14: Figure S8 Colinearity of common markers between our study (middle) and Bordat *et al*. ([39]; left) and Loridon *et al*. ([38]; right) composite maps. Click here for file

Additional file 15: Figure S9 Dot-plot of syntenic relationships between the *P. sativum* linkage groups (PsLG) and the *M. truncatula* pseudo-chromosomes (MtrChr). 1252 cDNA Pea contigs are placed on the dot-plot according to the position of their SNPs on the pea LG (x-axis) and the position of their best blasts hits on the *M. truncatula* pseudo-chromosomes y-axis). Synteny conservation is observed when homolog points are placed on diagonal lines and block inversions when homolog points are perpendicular to this diagonal. Click here for file

Additional file 16: Figure S11 Collinear positions between the *P.sativum* LGVI and LGIII composite genetic map and *M. truncatula* Mtchr6, Mtchr2 and Mtchr3 physical map. Click here for file

Additional file 17: Table S6 List of Accessions used for sequencing and/or genotyping. Click here for file
