# Supplementary figures and images for: Transcriptome sequencing for high throughput SNP development and genetic mapping in Pea
Source: BMC Genomics. 2014 Feb 12;15:126. doi: 10.1186/1471-2164-15-126 (PMC3925251; doi:10.1186/1471-2164-15-126)

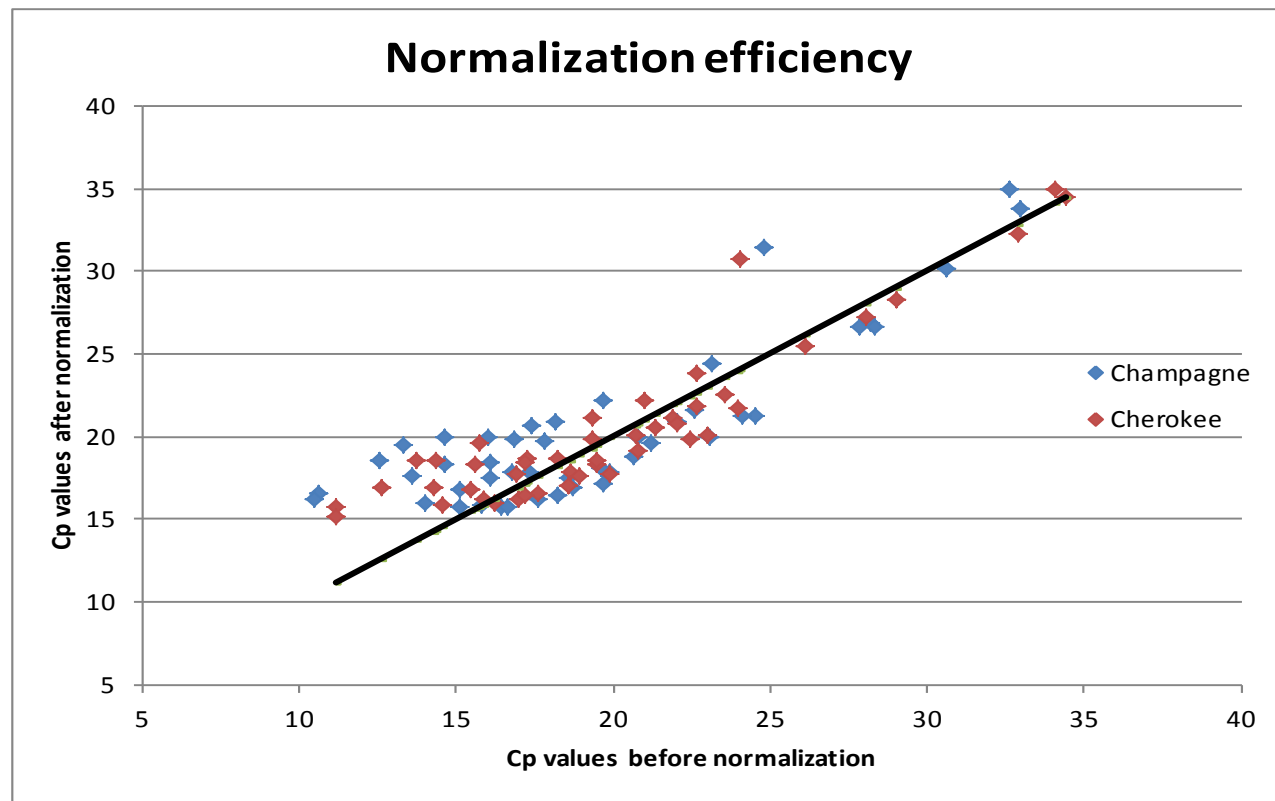

Supplement: Additional file 1: Figure S1 — Expression levels of 48 genes between initial (before normalization, X axis) and normalized (Y axis) conditions for the two genotypes Champagne (blue) and Cherokee (red). Expression level was assessed by Q-PCR and estimated by Cp (Crossing point), where high Cp indicates a low expression level, and low Cp a high expression level. [file 1471-2164-15-126-S1.pdf]

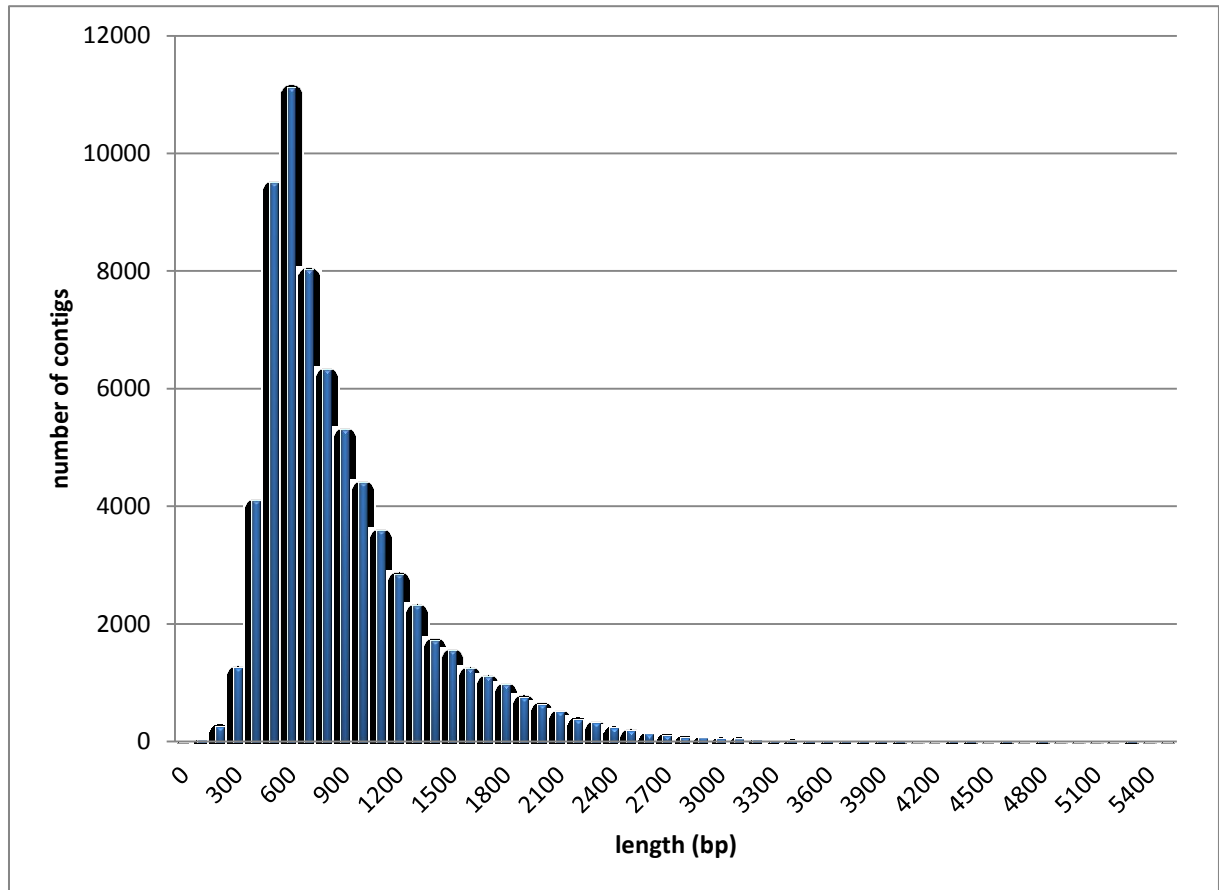

Supplement: Additional file 2: Figure S2 — Length distribution of the 68,850 contigs resulting from the de novo assembly of 454 sequencing data from 8 pea genotypes. [file 1471-2164-15-126-S2.pdf]

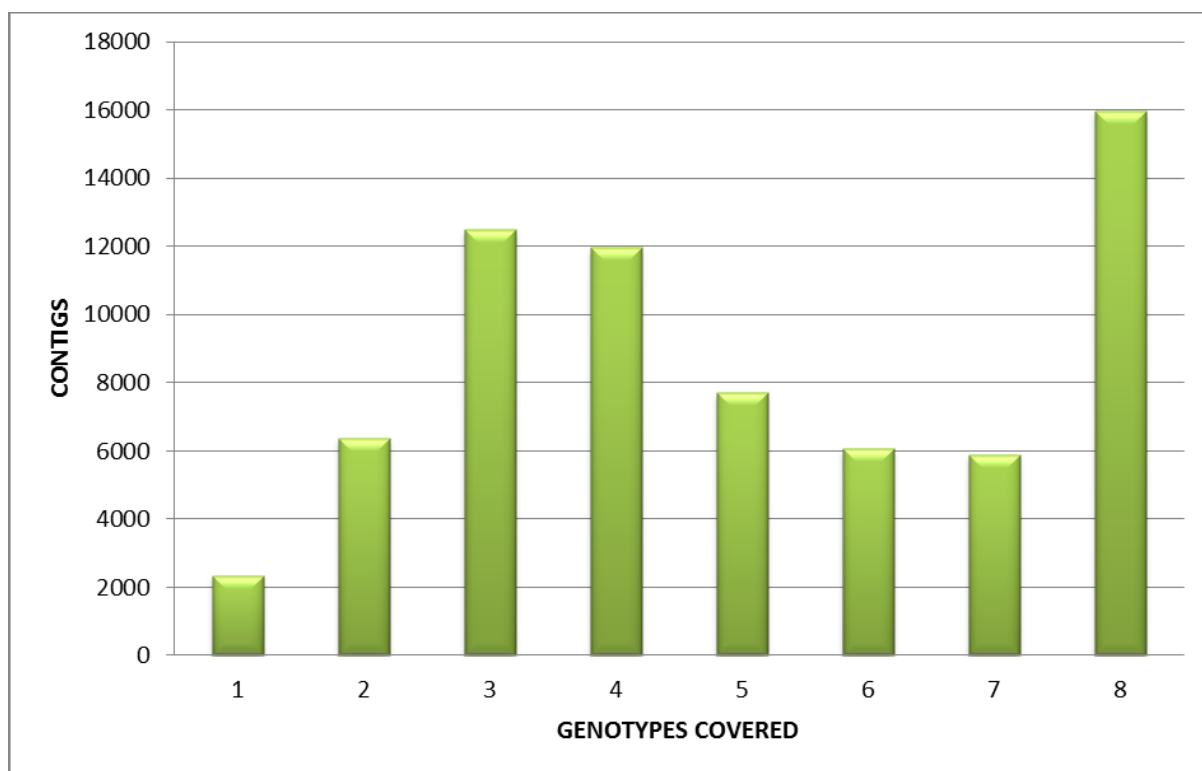

Supplement: Additional file 3: Figure S3 — Distribution of the pea genotypes’ contribution to the 68,850 contigs. [file 1471-2164-15-126-S3.pdf]

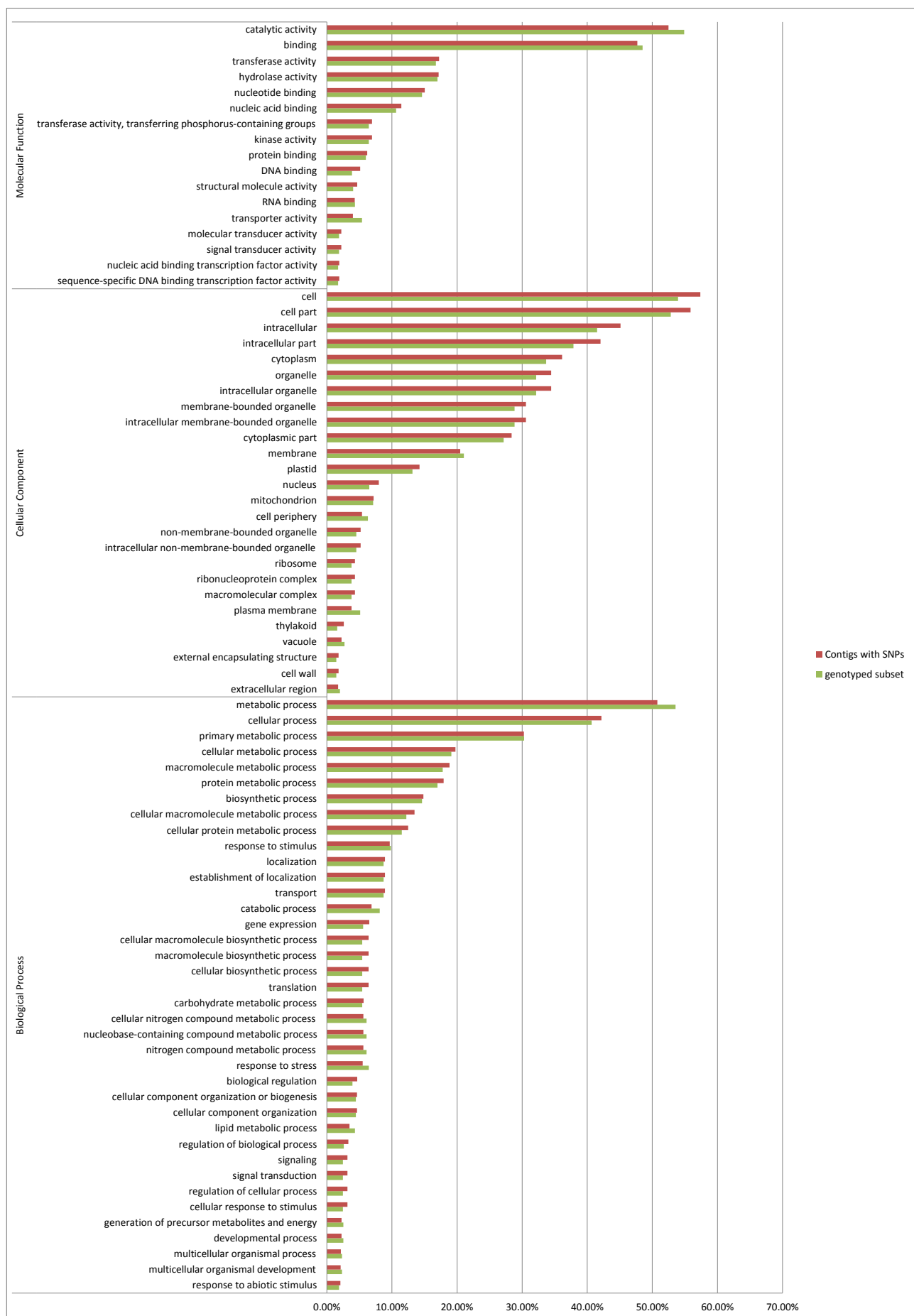

Supplement: Additional file 5: Figure S4 — GO term distribution comparison between the 7,338 annotated contigs set (from the 10,522 contigs containing robust SNPs, orange bars, only terms present in more than 1% of contigs shown) and the 1,920 subset that was genotyped (green bars). [file 1471-2164-15-126-S5.pdf]

Factorial analysis: Axes 1 / 2

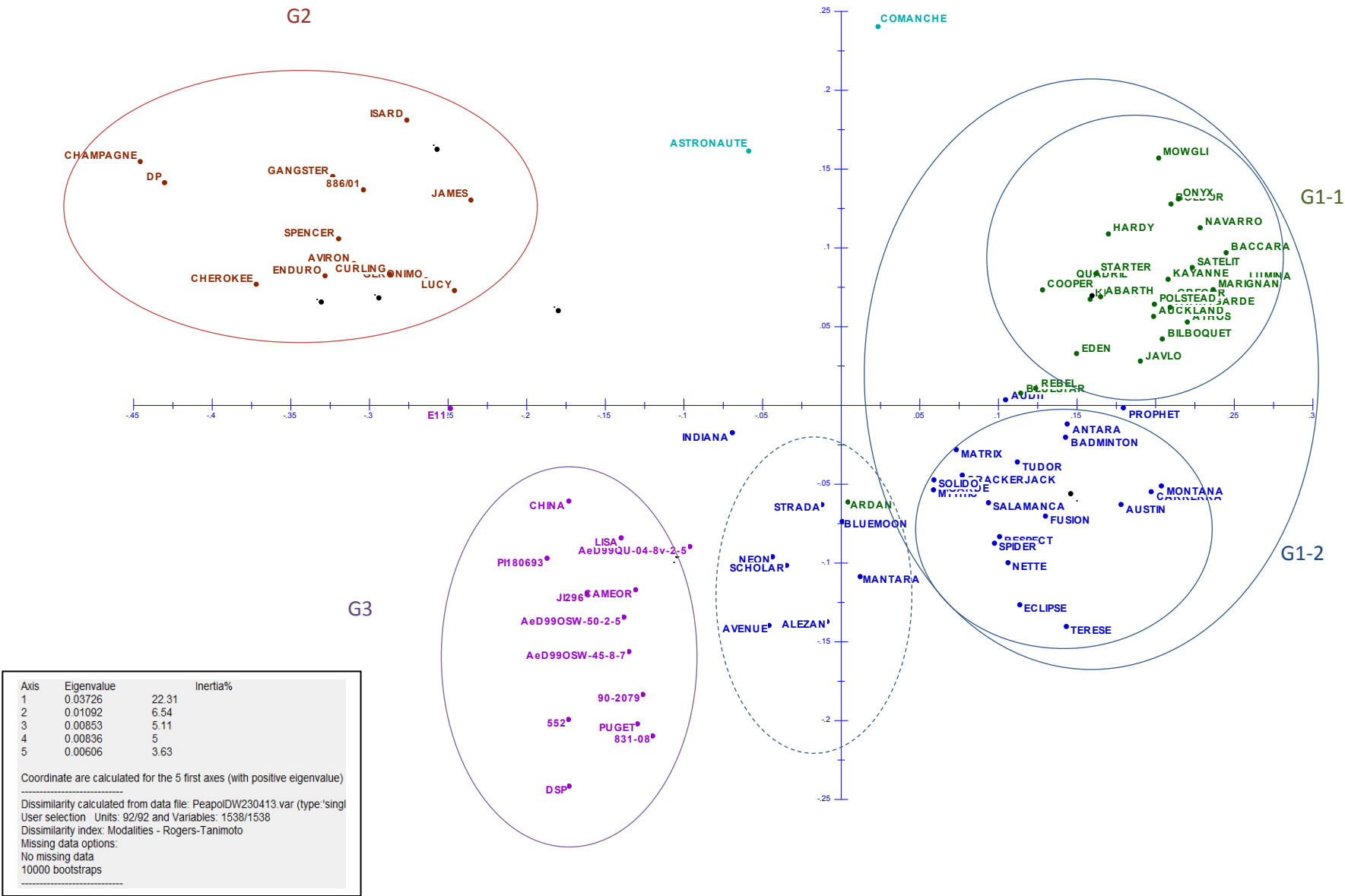

Supplement: Additional file 10: Figure S5 — Groupings across the 92 accessions and cultivars diversity panel revealed by a Factorial Analysis on genotyping data from 1538 SNP markers. [file 1471-2164-15-126-S10.pdf]

Hierarchical Clustering  
With 297 SNP

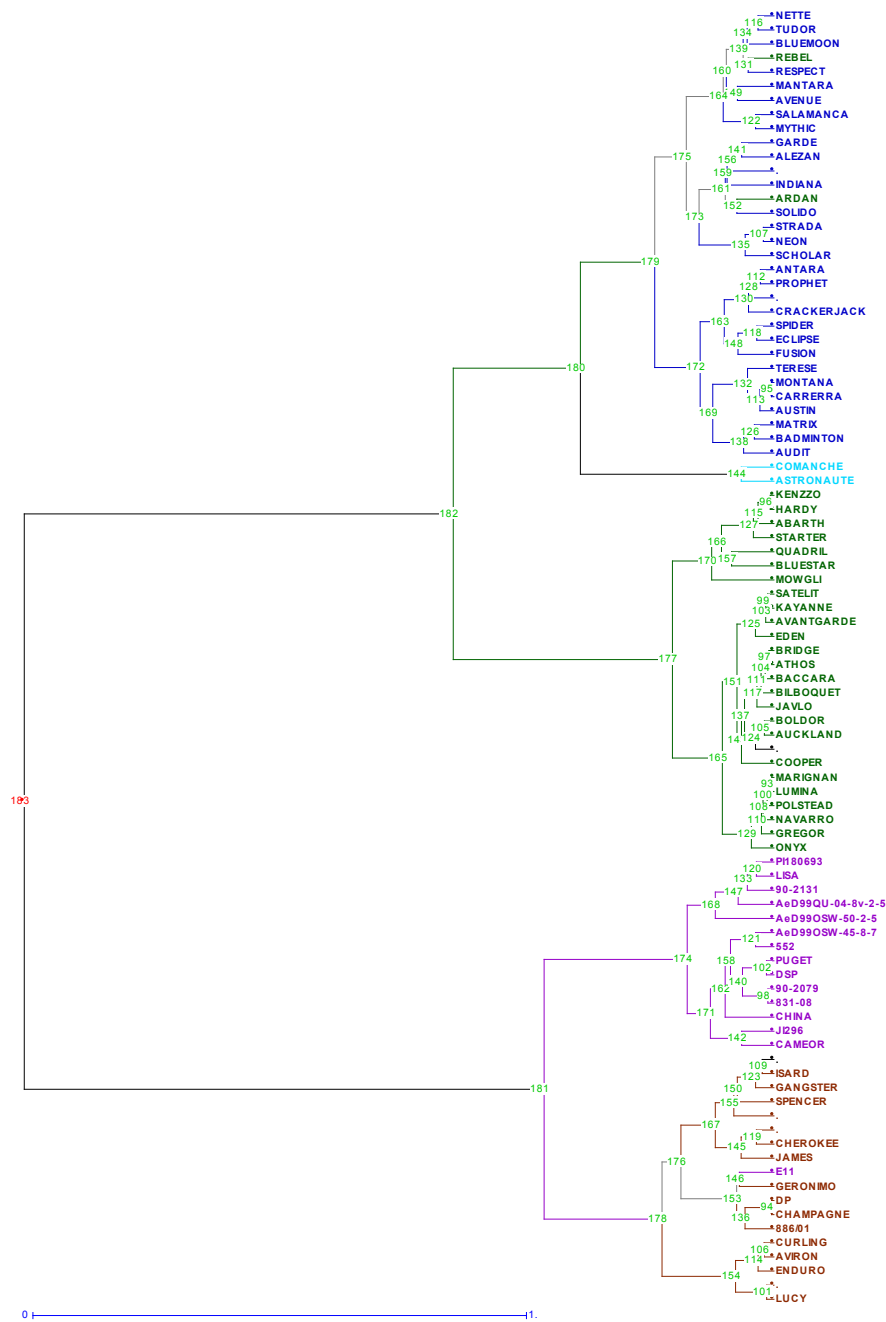

Supplement: Additional file 11: Figure S6 — Classification of a diversity panel of 92 pea accessions using 297 SNPs. Rogers’ distances were computed for all pairs of accessions and a Ward hierarchical classification procedure was used to classify the accessions in clusters (Cx) and subclusters (Cx-x). [file 1471-2164-15-126-S11.pdf]

# LGI

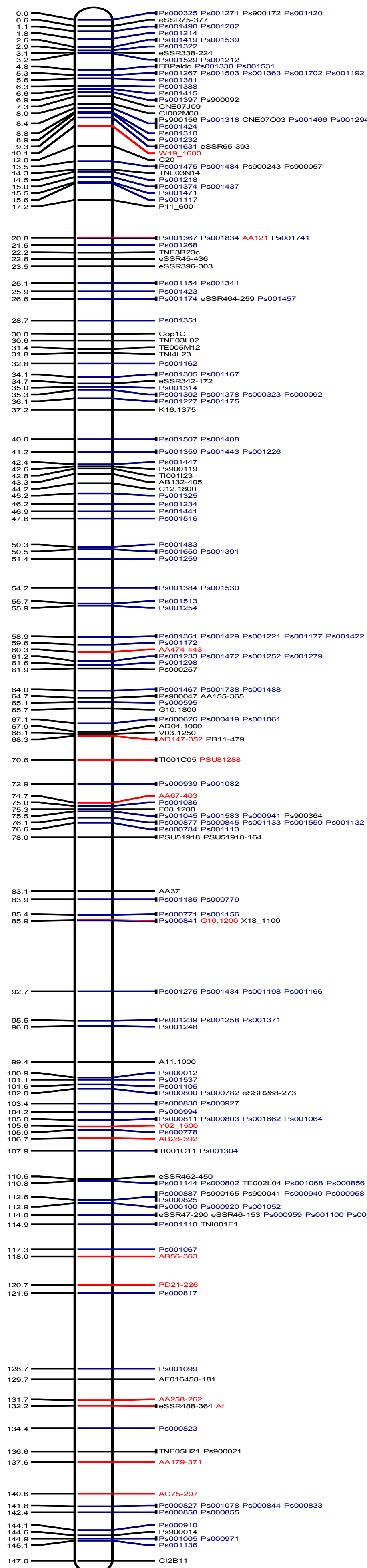

# LGII

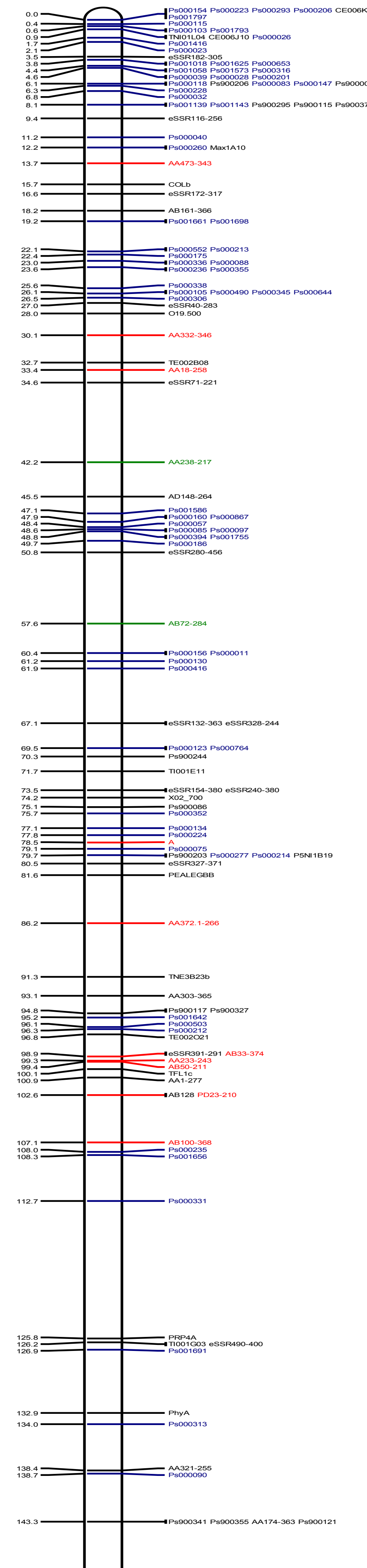

# LGIII

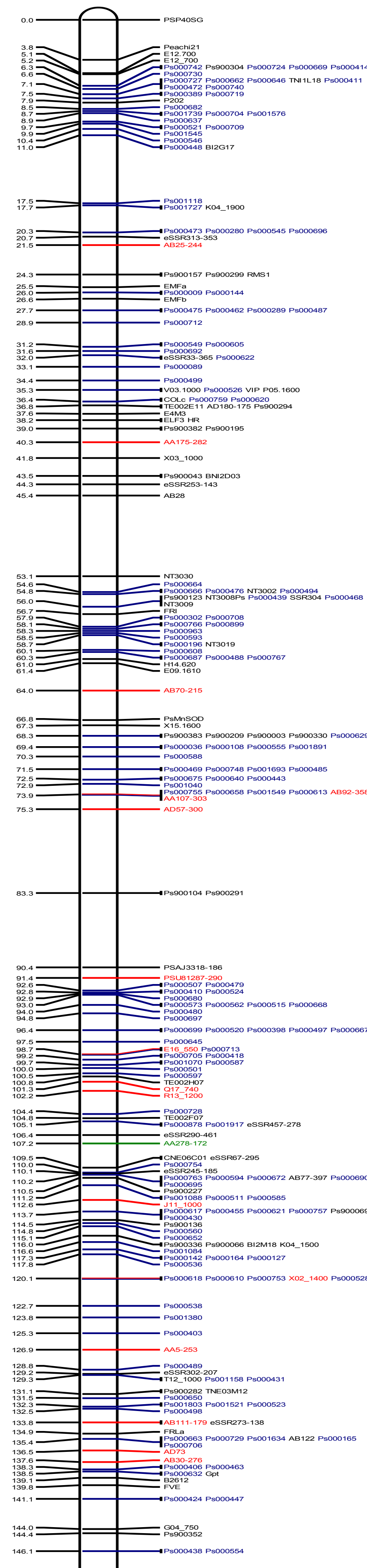

# LGIV

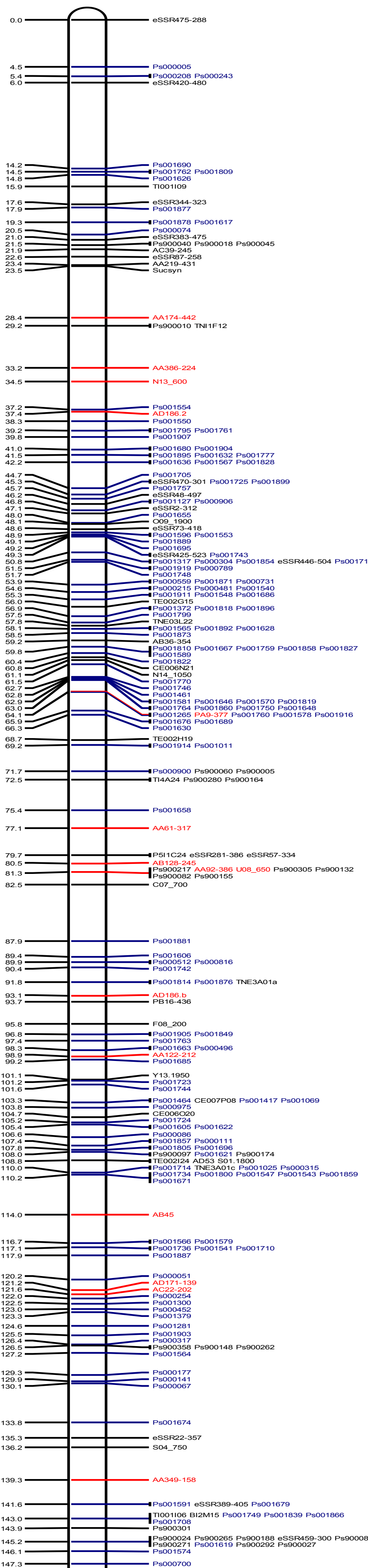

# LGV

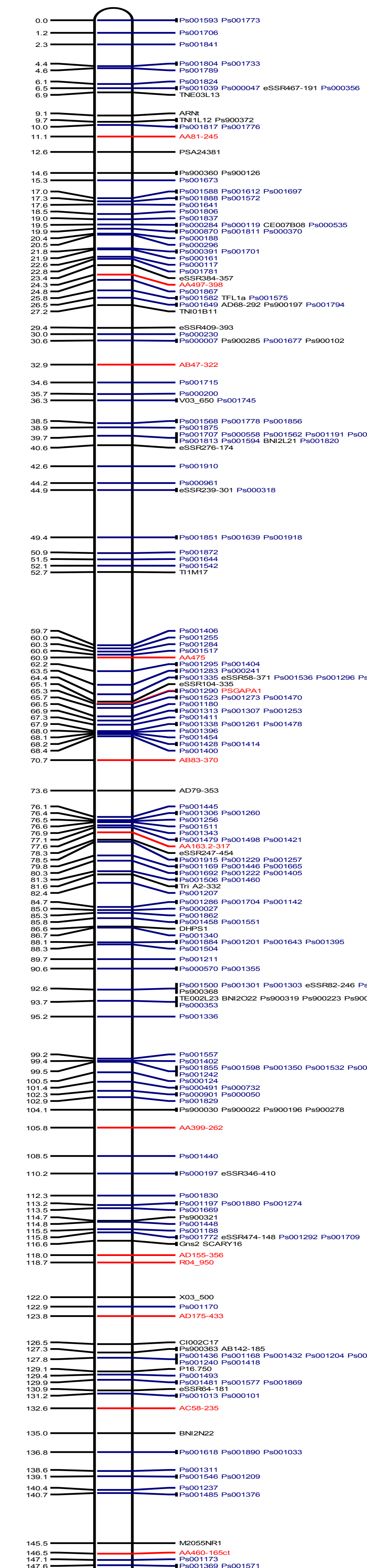

# LGVI

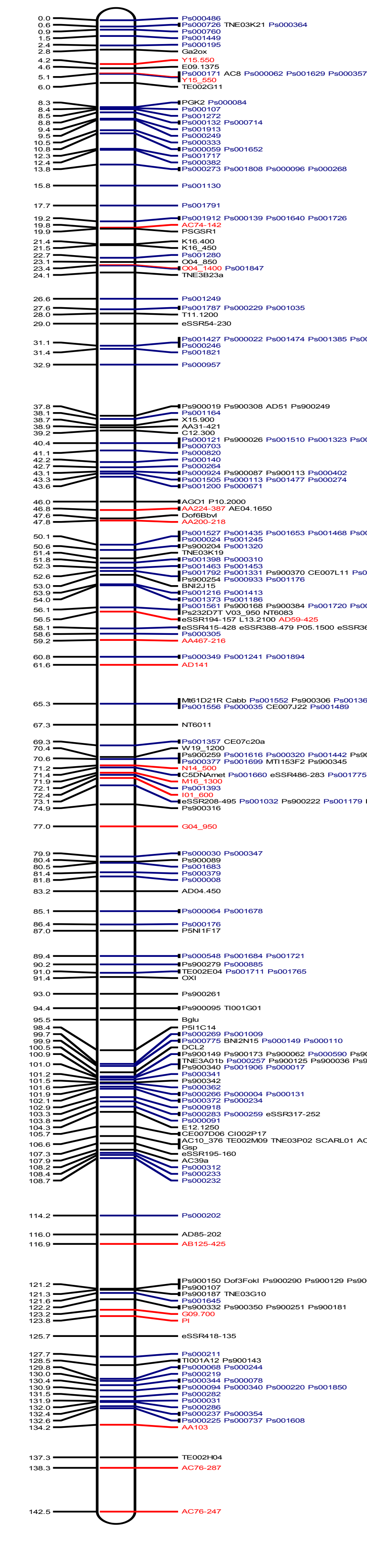

# LGVII

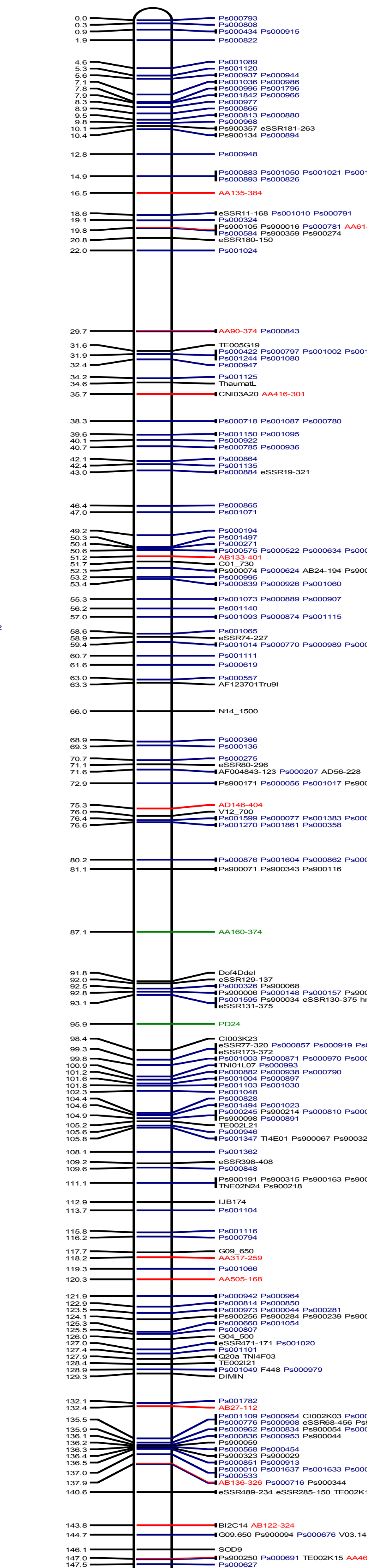

Supplement: Additional file 12: Figure S7 — P. sativum composite map presenting 1340 newly developed SNP markers (shown in blue). Most markers shown in red are SSR markers common with a previous consensus map (Loridon et al.[38]). Distances are in cM (Haldane). [file 1471-2164-15-126-S12.pdf]

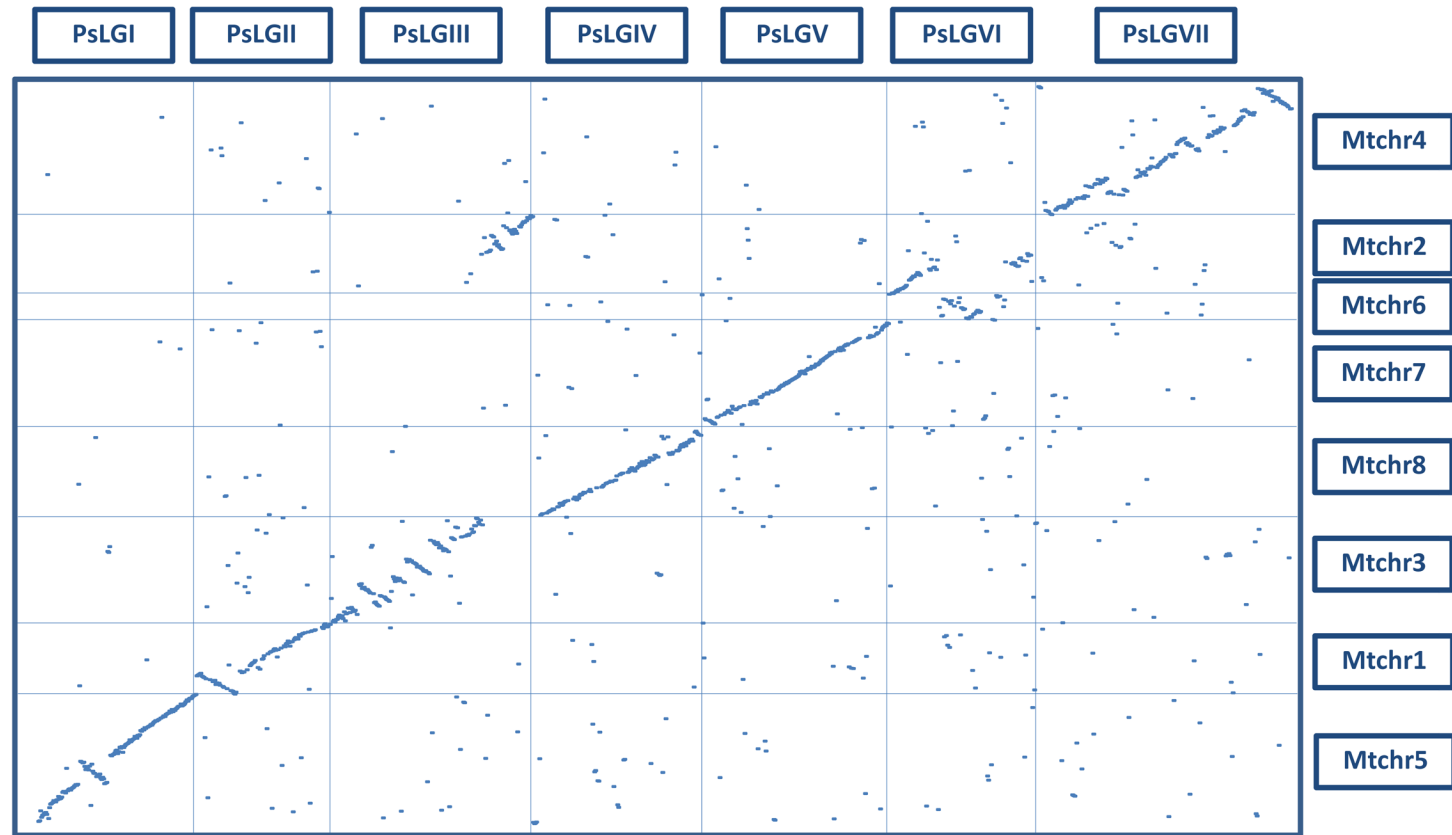

Supplement: Additional file 15: Figure S9 — Dot-plot of syntenic relationships between the P. sativum linkage groups (PsLG) and the M. truncatula pseudo-chromosomes (MtrChr). 1252 cDNA Pea contigs are placed on the dot-plot according to the position of their SNPs on the pea LG (x-axis) and the position of their best blasts hits on the M. truncatula pseudo-chromosomes y-axis). Synteny conservation is observed when homolog points are placed on diagonal lines and block inversions when homolog points are perpendicular to this diagonal. [file 1471-2164-15-126-S15.pdf]

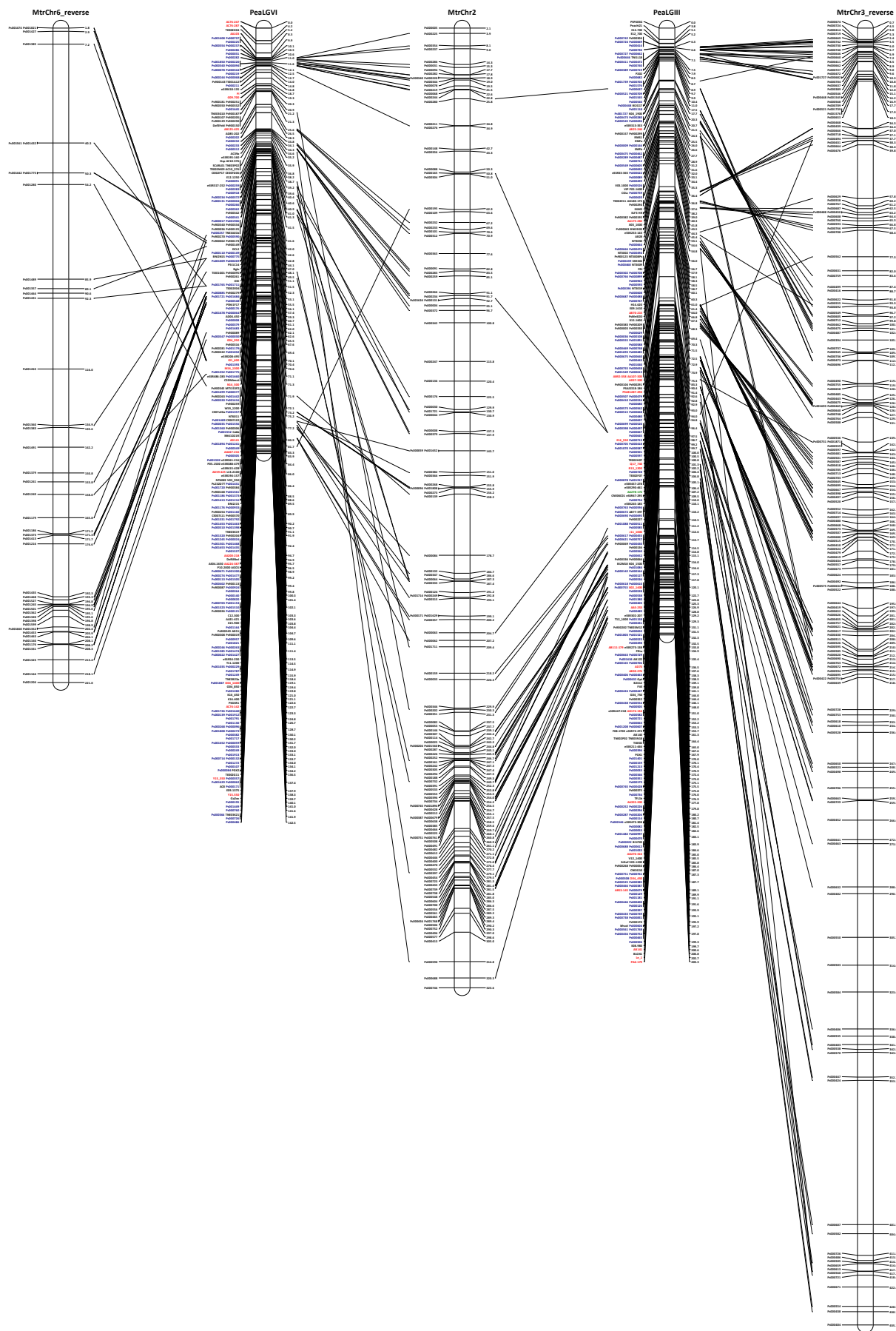

Supplement: Additional file 16: Figure S11 — Collinear positions between the P.sativum LGVI and LGIII composite genetic map and M. truncatula Mtchr6, Mtchr2 and Mtchr3 physical map. [file 1471-2164-15-126-S16.pdf]
