# Supplementary material for: Transcriptome sequencing for high throughput SNP development and genetic mapping in Pea
Source: BMC Genomics. 2014 Feb 12;15:126. doi: 10.1186/1471-2164-15-126 (PMC3925251; doi:10.1186/1471-2164-15-126)
Supplement: Additional file 7: Table S3 — Fisher’s Exact Test with Multiple Testing Correction of FDR (Benjamini and Hochberg). Significantly differentially represented terms from comparing a test group (1920 contigs subset) to a reference group (10,522 contigs set) for Gene Ontology terms enrichment. [file 1471-2164-15-126-S7.pdf]

GO Term	Name	Type	FDR	single test p-Value	# in test group	# in reference group	# non annot test	# non annot reference group	Over/Under
GO:0005215	transporter activity	F	6,5E-1	2,2E-2	81	233	1424	5600	over
GO:0009579	thylakoid	C	6,5E-1	2,3E-2	24	152	1481	5681	under
GO:0005886	plasma membrane	C	6,5E-1	2,3E-2	77	221	1428	5612	over
GO:0040007	growth	P	8,2E-1	3,9E-2	11	19	1494	5814	over
GO:0003677	DNA binding	F	8,2E-1	4,4E-2	58	298	1447	5535	under
