# Supplementary material for: Transcriptome sequencing for high throughput SNP development and genetic mapping in Pea
Source: BMC Genomics. 2014 Feb 12;15:126. doi: 10.1186/1471-2164-15-126 (PMC3925251; doi:10.1186/1471-2164-15-126)
Supplement: Additional file 9: Table S5 — SNP Quality criteria. [file 1471-2164-15-126-S9.pdf]

Table S5: SNPs Quality criteria

Quality	% Heterozygous	% Failed datas	Signal Intensity R	Theta T
**A**	*<10%*	*<5%*	*AA R Mean AND AB R Mean AND BB R Mean >0,2*	*AA T Mean < 0,2 AND BB T Mean > 0,8*
**B**	*<10%*	*<5%*	*AA R Mean OU AB R Mean OU BB R Mean <0,20*	*AA T Mean < 0,2 AND BB T Mean > 0,8*
**C**	*<10%*	*<5%*	*ALL DATAS*	*AA T Mean > 0,2 OR BB T Mean < 0,8 OR AB T Mean < 0,2 OR > 0,8*
**D**	*<10%*	*>5%*	*ALL DATAS*	*ALL DATAS*
**E**	*FAILED*	*FAILED*	*FAILED*	*FAILED*
**H**	*> 10%*	ALL DATAS	ALL DATAS	ALL DATAS
