# Supplementary material for: Transcriptome sequencing for high throughput SNP development and genetic mapping in Pea
Source: BMC Genomics. 2014 Feb 12;15:126. doi: 10.1186/1471-2164-15-126 (PMC3925251; doi:10.1186/1471-2164-15-126)
Supplement: Additional file 13: Figure S10 — Collinear positions between the P.sativum composite genetic map and M. truncatula physical map. For pea linkage groups, 1u = 1 cM; for M. truncatula pseudo-chromosomes, 1u = 0.1 Mb. [file 1471-2164-15-126-S13.pdf]

Figure S10: Collinearity of common markers between our study (2013; middle) Bordat *et al.* (2011; left) Loridon *et al.* (2005; right) composite maps

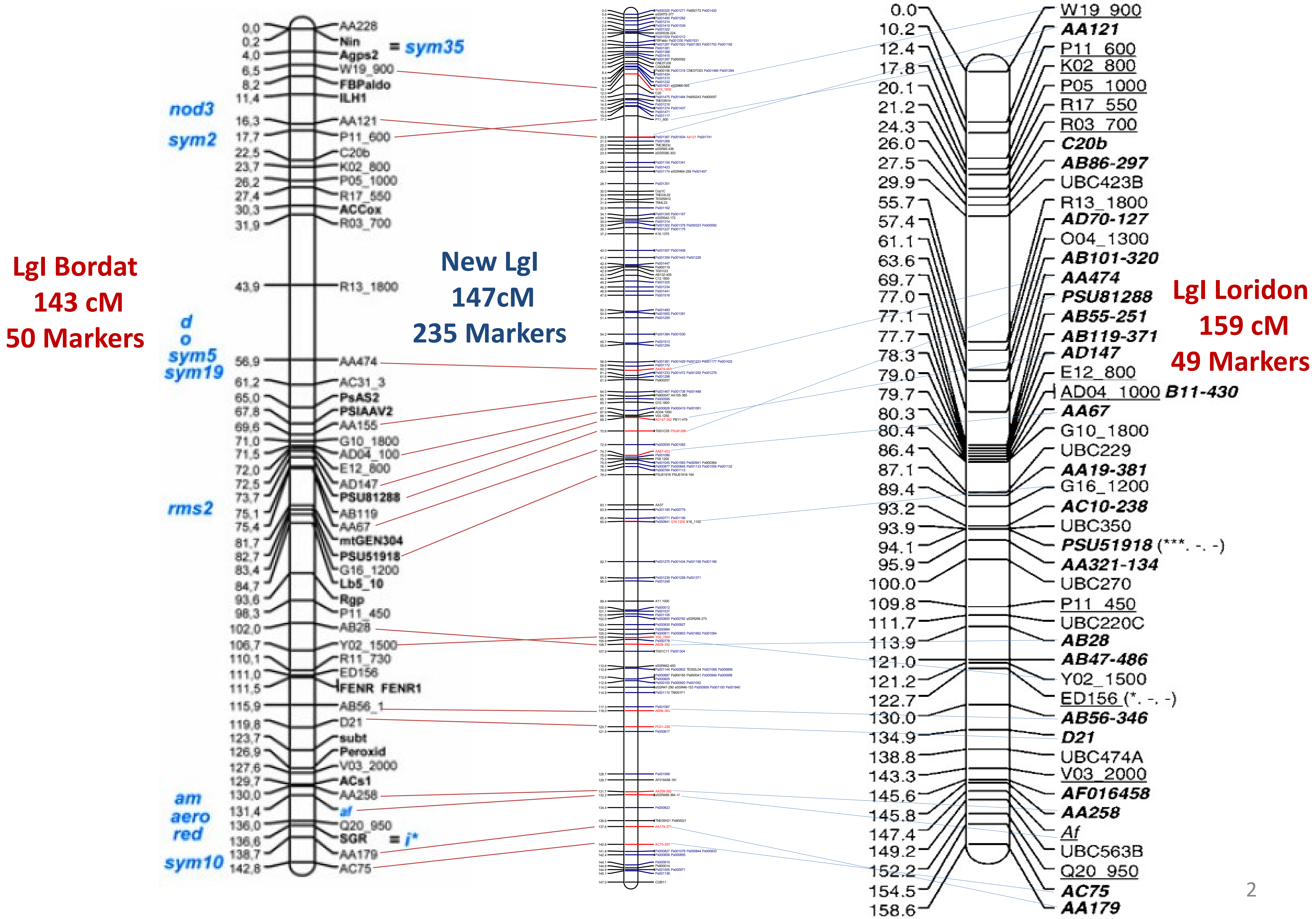
LgI Bordat
143 cM
50 Markers
New LgI
147cM
235 Markers
LgI Loridon
159 cM
49 Markers
0.0 AA228
0.2 Nin
4.0 Agps2
6.5 W19_900
8.2 FBPaldo
11.4 ILH1
16.3 AA121
17.7 P11_600
22.5 C20b
23.7 K02_800
26.2 P05_1000
27.4 R17_550
30.3 ACCox
31.9 R03_700
43.9 R13_1800
56.9 AA474
61.2 AC31_3
65.0 PsAS2
67.8 PSIAAV2
69.6 AA155
71.0 G10_1800
71.5 AD04_100
72.0 E12_800
72.5 AD147
73.7 PSU81288
75.1 AB119
75.4 AA67
81.7 mtGEN304
82.7 PSU51918
83.4 G16_1200
84.7 Lb5_10
93.6 Rgp
98.3 P11_450
102.0 AB28
106.7 Y02_1500
110.1 R11_730
111.0 ED156
111.5 FENR FENR1
115.9 AB56_1
119.8 D21
123.7 subt
126.9 Peroxid
127.6 V03_2000
129.7 ACs1
130.0 AA258
131.4 af
136.0 Q20_950
136.6 SGR
138.7 AA179
142.8 AC75
= sym35
nod3
sym2
d
o
sym5
sym19
rms2
am
aero
red
sym10
= i*
0.0 W19_900
10.2 AA121
12.4 P11_600
17.8 K02_800
20.1 P05_1000
21.2 R17_550
24.3 R03_700
26.0 C20b
27.5 AB86-297
29.9 UBC423B
55.7 R13_1800
57.4 AD70-127
61.1 O04_1300
63.6 AB101-320
69.7 AA474
77.0 PSU81288
77.1 AB55-251
77.7 AB119-371
78.3 AD147
79.0 E12_800
79.7 AD04_1000 B11-430
80.3 AA67
80.4 G10_1800
86.4 UBC229
87.1 AA19-381
89.4 G16_1200
93.2 AC10-238
93.9 UBC350
94.1 PSU51918 (***. -. -)
95.9 AA321-134
100.0 UBC270
109.8 P11_450
111.7 UBC220C
113.9 AB28
121.0 AB47-486
121.2 Y02_1500
122.7 ED156 (*. -. -)
130.0 AB56-346
134.9 D21
138.8 UBC474A
143.3 V03_2000
145.6 AF016458
145.8 AA258
147.4 Af
149.2 UBC563B
152.2 Q20_950
154.5 AC75
158.6 AA179

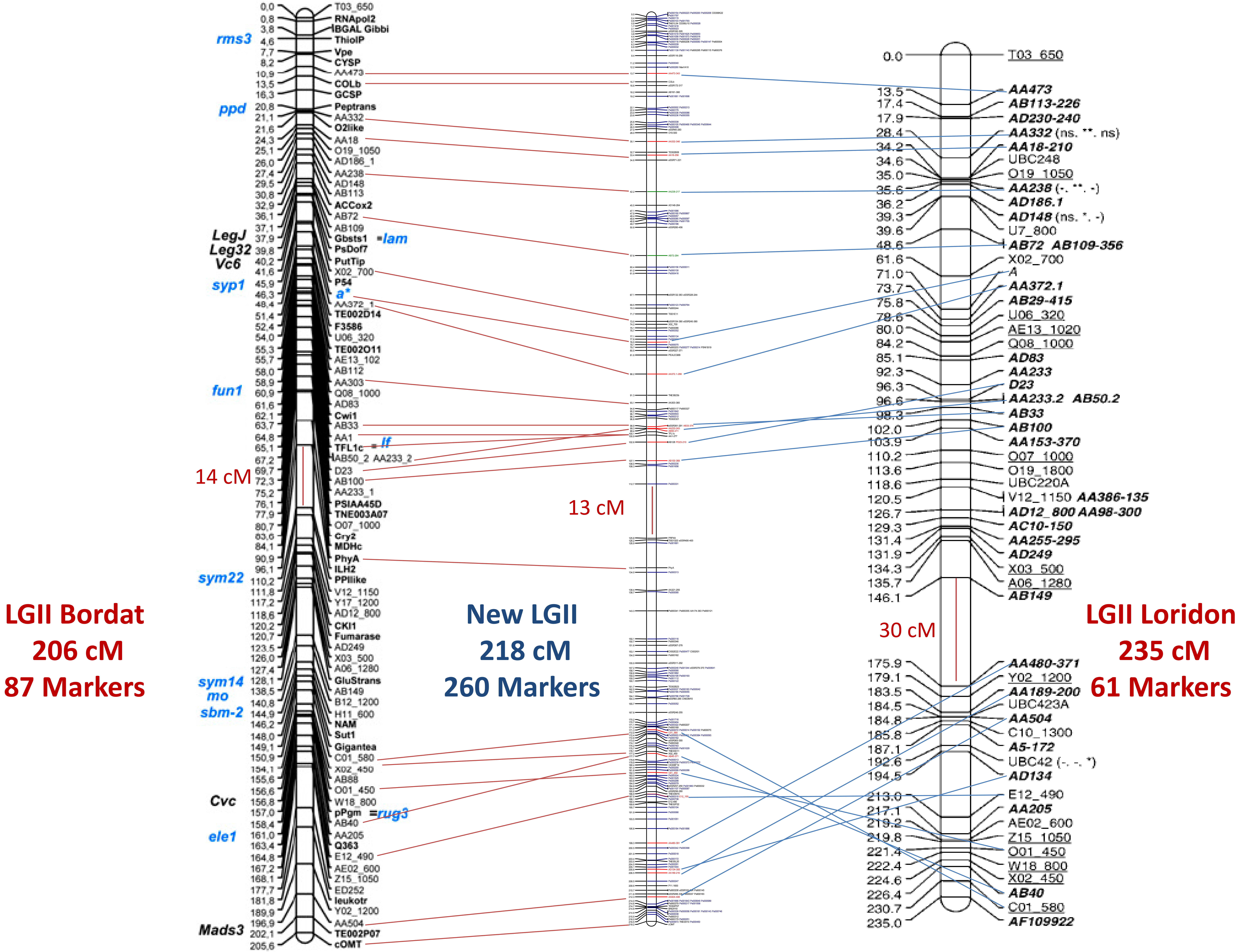

LGII Bordat
206 cM
87 Markers
New LGII
218 cM
260 Markers
LGII Loridon
235 cM
61 Markers
14 cM
13 cM
30 cM
rms3
ppd
LegJ
Leg32
Vc6
syp1
fun1
sym22
sym14
mo
sbm-2
Cvc
ele1
Mads3
=lam
a*
=lf
=rug3
T03_650
RNApol2
BGAL Gibbi
ThiolP
Vpe
CYSP
AA473
COLb
GCSP
Peptrans
AA332
O2like
AA18
O19_1050
AD186_1
AA238
AD148
AB113
ACCox2
AB72
AB109
Gbsts1
PsDof7
PutTip
X02_700
P54
AA372_1
TE002D14
F3586
U06_320
TE002O11
AE13_102
AB112
AA303
Q08_1000
AD83
Cwi1
AB33
AA1
TFL1c
AB50_2 AA233_2
D23
AB100
AA233_1
PSIAA45D
TNE003A07
O07_1000
Cry2
MDHc
PhyA
ILH2
PPIlike
V12_1150
Y17_1200
AD12_800
CKI1
Fumarase
AD249
X03_500
A06_1280
GluStrans
AB149
B12_1200
H11_600
NAM
Sut1
Gigantea
C01_580
X02_450
AB88
O01_450
W18_800
pPgm
AB40
AA205
Q363
E12_490
AE02_600
Z15_1050
ED252
leukotr
Y02_1200
AA504
TE002P07
cOMT
T03_650
AA473
AB113-226
AD230-240
AA332 (ns. **. ns)
AA18-210
UBC248
O19_1050
AA238 (-. **. -)
AD186.1
AD148 (ns. *. -)
U7_800
AB72 AB109-356
X02_700
A
AA372.1
AB29-415
U06_320
AE13_1020
Q08_1000
AD83
AA233
D23
AA233.2 AB50.2
AB33
AB100
AA153-370
O07_1000
O19_1800
UBC220A
V12_1150 AA386-135
AD12_800 AA98-300
AC10-150
AA255-295
AD249
X03_500
A06_1280
AB149
AA480-371
Y02_1200
AA189-200
UBC423A
AA504
C10_1300
A5-172
UBC42 (-. -. *)
AD134
E12_490
AA205
AE02_600
Z15_1050
O01_450
W18_800
X02_450
AB40
C01_580
AF109922

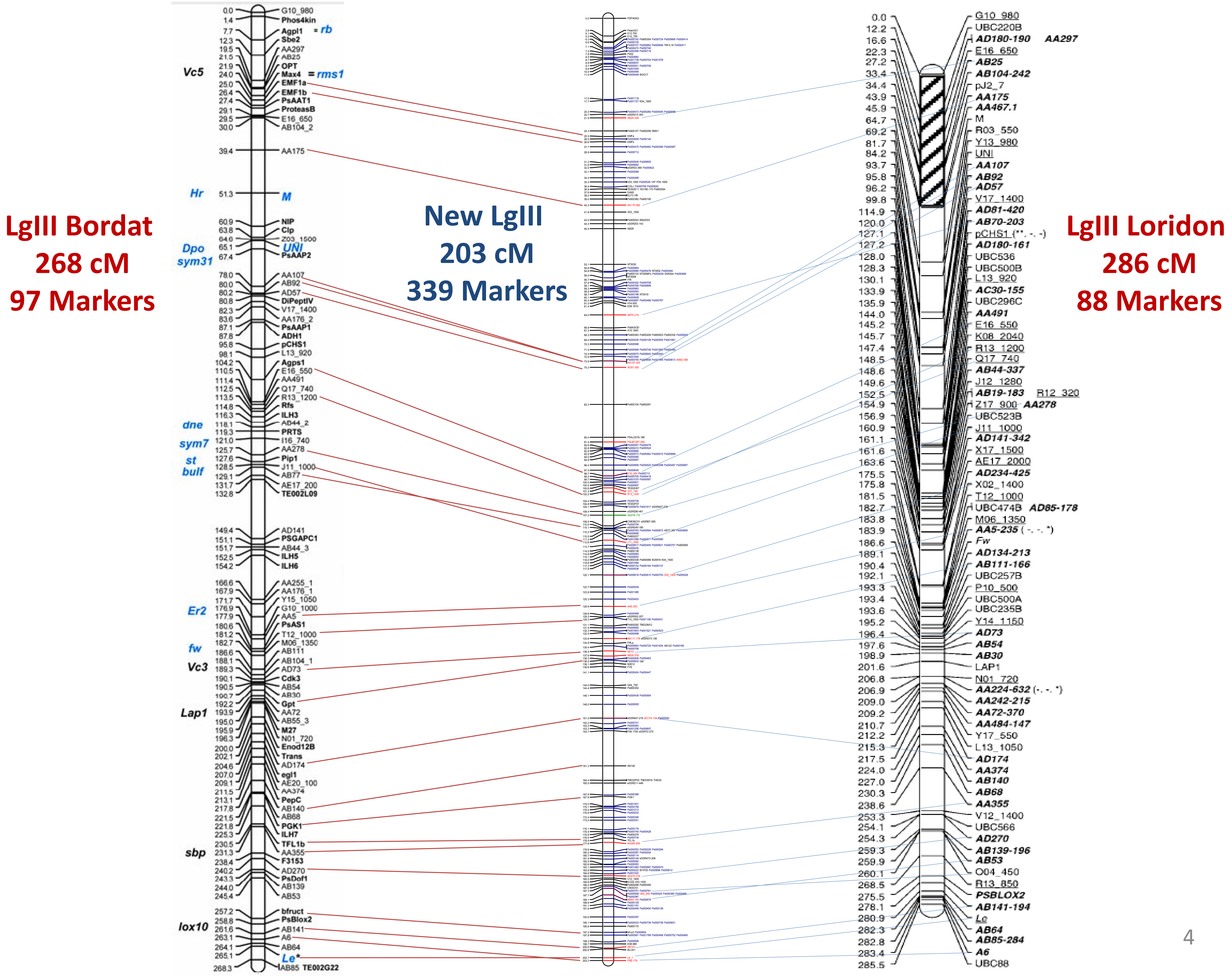
LgIII Bordat
268 cM
97 Markers
New LgIII
203 cM
339 Markers
LgIII Loridon
286 cM
88 Markers
Vc5
Hr
Dpo
sym31
dne
sym7
st
bulf
Er2
fw
Vc3
Lap1
sbp
lox10
0.0 G10_980
1.4 Phos4kin
7.7 Agpl1 = rb
12.3 Sbe2
19.5 AA297
21.5 AB25
21.9 OPT
24.0 Max4 = rms1
25.0 EMF1a
26.4 EMF1b
27.4 PsAAT1
29.1 ProteasB
29.5 E16_650
30.0 AB104_2
39.4 AA175
51.3 M
60.9 NIP
63.8 Clp
64.6 Z03_1500
65.1 UNI
67.4 PsAAP2
78.0 AA107
80.0 AB92
80.2 AD57
80.8 DiPeptIV
82.3 V17_1400
83.6 AA176_2
87.1 PsAAP1
87.8 ADH1
95.8 pCHS1
98.1 L13_920
104.2 Agps1
110.5 E16_550
111.4 AA491
112.5 Q17_740
113.5 R13_1200
114.8 Rfs
116.3 ILH3
118.1 AD44_2
119.3 PRTS
121.0 I16_740
125.7 AA278
127.6 Pip1
128.5 J11_1000
129.1 AB77
131.7 AE17_200
132.8 TE002L09
149.4 AD141
151.1 PSGAPC1
151.7 AB44_3
152.5 ILH5
154.2 ILH6
166.6 AA255_1
167.9 AA176_1
171.7 Y15_1050
176.9 G10_1000
177.9 AA5
180.6 PsAS1
181.2 T12_1000
182.7 M06_1350
186.6 AB111
188.1 AB104_1
189.3 AD73
190.1 Cdk3
190.5 AB54
190.7 AB30
192.2 Gpt
193.9 AA72
195.0 AB55_3
195.9 M27
196.3 N01_720
200.0 Enod12B
202.1 Trans
204.6 AD174
207.0 egl1
209.1 AE20_100
211.5 AA374
213.1 PepC
217.8 AB140
221.5 AB68
221.8 PGK1
225.3 ILH7
230.5 TFL1b
231.3 AA355
238.4 F3153
240.2 AD270
243.3 PsDof1
244.0 AB139
245.4 AB53
257.2 bfruct
258.8 PsBlox2
261.6 AB141
263.1 A6
264.1 AB64
265.1 Le*
268.3 AB85 TE002G22
0.0 G10_980
12.2 UBC220B
16.6 AD180-190 AA297
22.3 E16_650
27.2 AB25
33.4 AB104-242
34.4 pJ2_7
43.9 AA175
45.9 AA467.1
64.7 M
69.2 R03_550
81.7 Y13_980
84.2 UNI
93.7 AA107
95.8 AB92
96.2 AD57
99.8 V17_1400
114.9 AD61-420
120.0 AB70-203
127.1 pCHS1 (**, -, -)
127.2 AD180-161
128.0 UBC536
128.3 UBC500B
130.1 L13_920
133.9 AC30-155
135.9 UBC296C
144.0 AA491
145.2 E16_550
145.7 K08_2040
147.4 R13_1200
148.5 Q17_740
148.6 AB44-337
149.6 J12_1280
152.5 AB19-183 R12_320
154.9 Z17_900 AA278
156.9 UBC523B
160.9 J11_1000
161.1 AD141-342
161.6 X17_1500
163.6 AE17_2000
175.5 AD234-425
175.8 X02_1400
181.5 T12_1000
182.7 UBC474B AD85-178
183.8 M06_1350
183.9 AA5-235 (-, -, *)
186.6 Fw
189.1 AD134-213
190.4 AB111-166
192.1 UBC257B
193.3 P10_500
193.4 UBC500A
193.6 UBC235B
195.2 Y14_1150
196.4 AD73
197.6 AB54
198.9 AB30
201.6 LAP1
206.8 N01_720
206.9 AA224-632 (-, -, *)
209.0 AA242-215
209.2 AA72-370
210.7 AA484-147
212.2 Y17_550
215.3 L13_1050
217.5 AD174
224.0 AA374
227.0 AB140
230.3 AB68
238.6 AA355
253.3 V12_1400
254.1 UBC566
254.3 AD270
259.3 AB139-196
259.9 AB53
260.1 O04_450
268.5 R13_850
275.5 PSBLOX2
278.1 AB141-194
280.9 Le
282.3 AB64
282.8 AB85-284
283.4 A6
285.5 UBC88

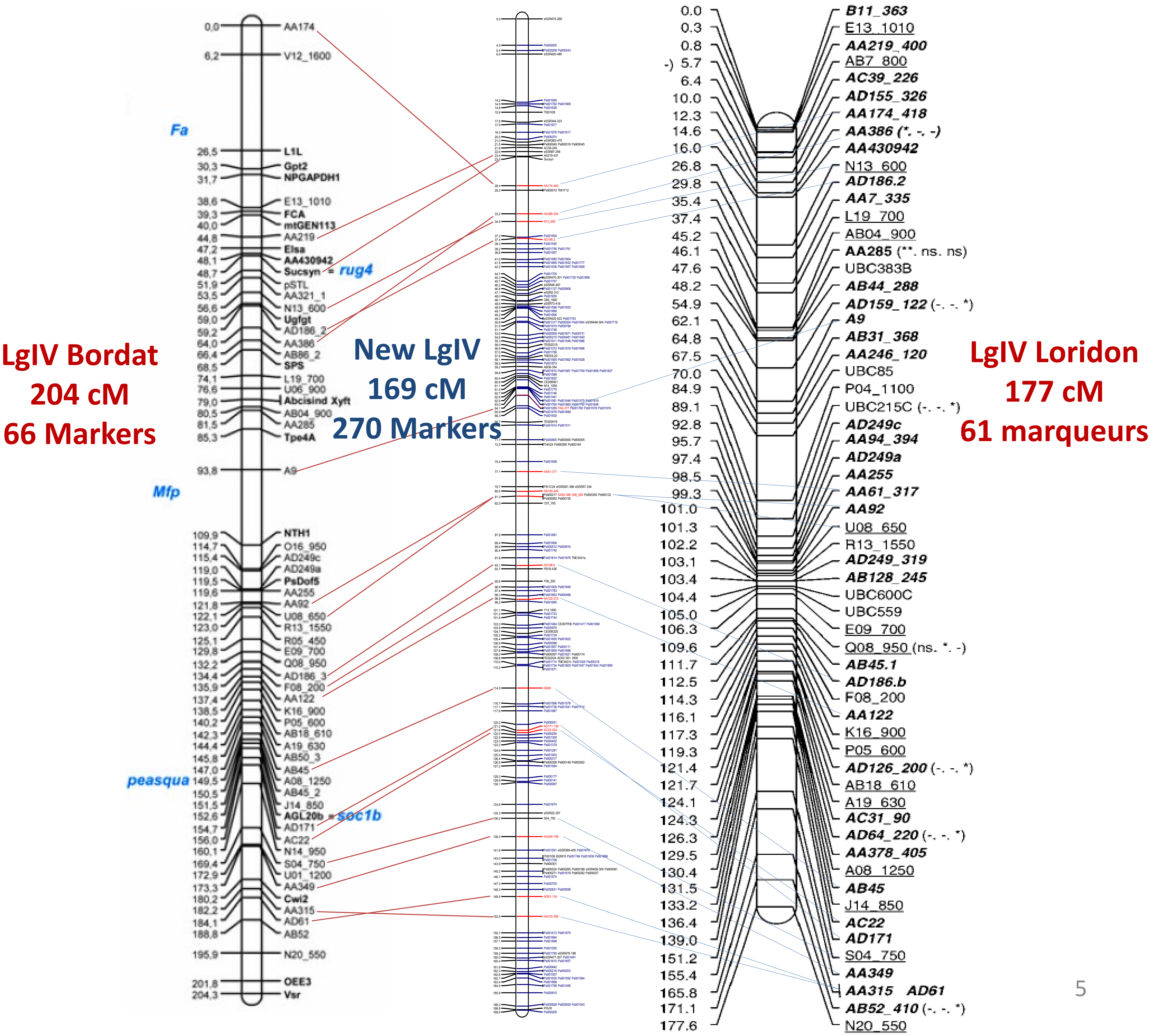

LgIV Bordat
204 cM
66 Markers
New LgIV
169 cM
270 Markers
LgIV Loridon
177 cM
61 marqueurs
Fa
Mfp
peasqua
0,0 AA174
6,2 V12_1600
26,5 L1L
30,3 Gpt2
31,7 NPGAPDH1
38,6 E13_1010
39,3 FCA
40,0 mtGEN113
44,8 AA219
47,2 Elsa
48,1 AA430942
48,7 Sucsyn = rug4
51,9 pSTL
53,5 AA321_1
56,6 N13_600
59,0 Ugfgt
59,2 AD186_2
64,0 AA386
66,4 AB86_2
68,5 SPS
74,1 L19_700
76,6 U06_900
79,0 Abcisind Xyft
80,5 AB04_900
81,5 AA285
85,3 Tpo4A
93,8 A9
109,9 NTH1
114,7 O16_950
115,4 AD249c
119,0 AD249a
119,5 PsDof5
119,6 AA255
121,8 AA92
122,1 U08_650
123,0 R13_1550
125,1 R05_450
129,8 E09_700
132,2 Q08_950
134,4 AD186_3
135,9 F08_200
137,4 AA122
138,5 K16_900
140,2 P05_600
142,3 AB18_610
144,4 A19_630
145,8 AB50_3
147,0 AB45
149,5 A08_1250
150,5 AB45_2
151,5 J14_850
152,6 AGL20b = soc1b
154,7 AD171
156,0 AC22
160,1 N14_950
169,4 S04_750
172,9 U01_1200
173,3 AA349
180,2 Cwi2
182,2 AA315
184,1 AD61
188,8 AB52
195,9 N20_550
201,8 OEE3
204,3 Vsr
0.0 B11_363
0.3 E13_1010
0.8 AA219_400
-) 5.7 AB7_800
6.4 AC39_226
10.0 AD155_326
12.3 AA174_418
14.6 AA386 (*. -. -)
16.0 AA430942
26.8 N13_600
29.8 AD186.2
35.4 AA7_335
37.4 L19_700
45.2 AB04_900
46.1 AA285 (**. ns. ns)
47.6 UBC383B
48.2 AB44_288
54.9 AD159_122 (-. -. *)
62.1 A9
64.8 AB31_368
67.5 AA246_120
70.0 UBC85
84.9 P04_1100
89.1 UBC215C (-. -. *)
92.8 AD249c
95.7 AA94_394
97.4 AD249a
98.5 AA255
99.3 AA61_317
101.0 AA92
101.3 U08_650
102.2 R13_1550
103.1 AD249_319
103.4 AB128_245
104.4 UBC600C
105.0 UBC559
106.3 E09_700
109.6 Q08_950 (ns. *. -)
111.7 AB45.1
112.5 AD186.b
114.3 F08_200
116.1 AA122
117.3 K16_900
119.3 P05_600
121.4 AD126_200 (-. -. *)
121.7 AB18_610
124.1 A19_630
124.3 AC31_90
126.3 AD64_220 (-. -. *)
129.5 AA378_405
130.4 A08_1250
131.5 AB45
133.2 J14_850
136.4 AC22
139.0 AD171
151.2 S04_750
155.4 AA349
165.8 AA315 AD61
171.1 AB52_410 (-. -. *)
177.6 N20_550

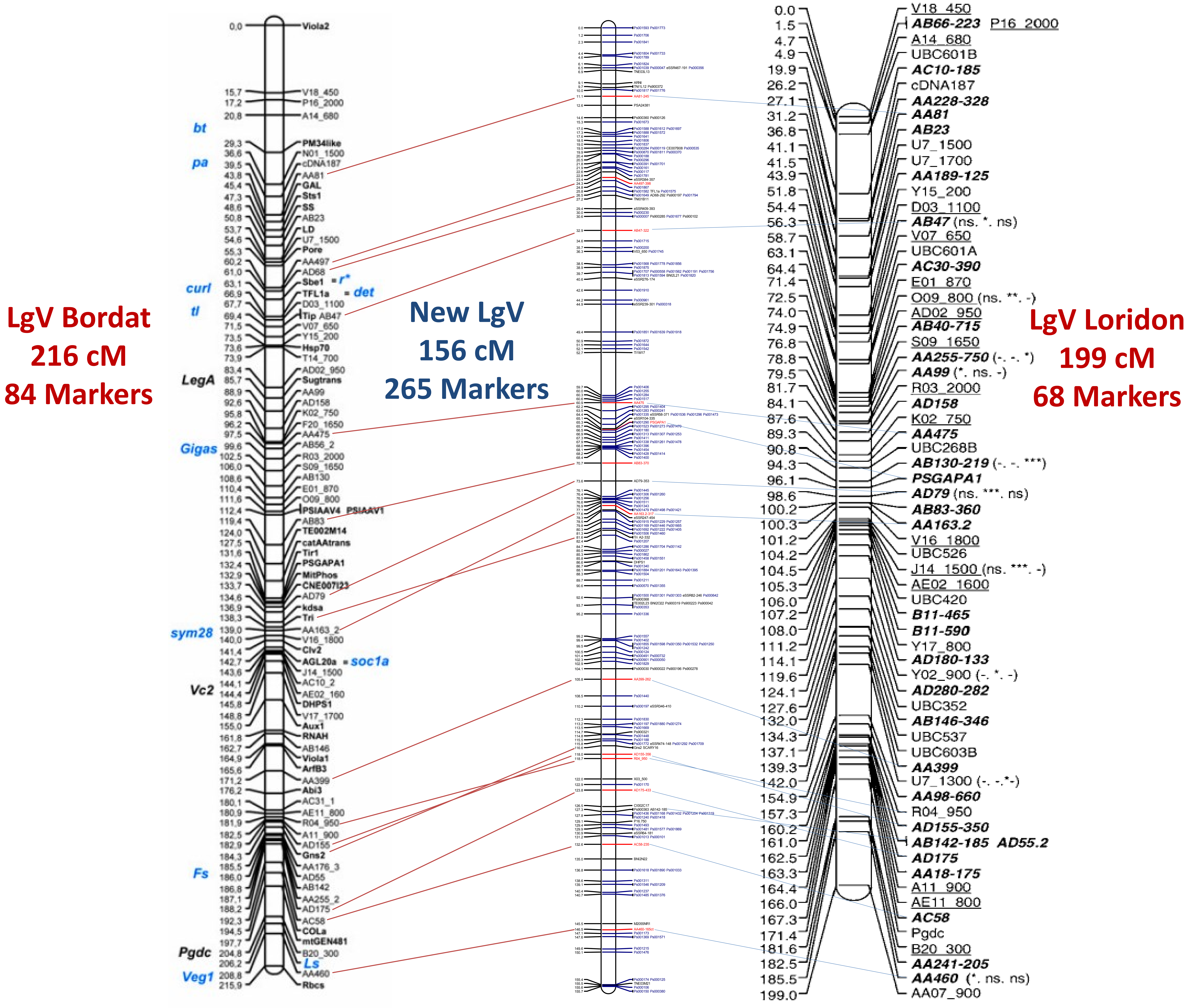
LgV Bordat
216 cM
84 Markers
New LgV
156 cM
265 Markers
LgV Loridon
199 cM
68 Markers
bt
pa
curl
tl
LegA
Gigas
sym28
Vc2
Fs
Pgdc
Veg1
0.0 Viola2
15.7 V18_450
17.2 P16_2000
20.8 A14_680
29.3 PM34like
36.6 N01_1500
39.5 cDNA187
43.8 AA81
45.4 GAL
47.3 Sts1
48.6 SS
50.8 AB23
53.7 LD
54.6 U7_1500
55.3 Pore
60.2 AA497
61.0 AD68
63.1 Sbe1 = r*
66.9 TFL1a = det
67.7 D03_1100
69.4 Tip AB47
71.5 V07_650
73.5 Y15_200
73.6 Hsp70
73.9 T14_700
83.4 AD02_950
85.7 Sugtrans
88.9 AA99
92.6 AD158
95.8 K02_750
96.2 F20_1650
97.5 AA475
99.6 AB56_2
102.5 R03_2000
106.0 S09_1650
108.6 AB130
110.4 E01_870
111.6 O09_800
112.4 PSIAAV4 PSIAAV1
119.4 AB83
124.0 TE002M14
127.5 catAAtrans
131.6 Tir1
132.4 PSGAPA1
132.9 MitPhos
133.7 CNE007I23
134.6 AD79
136.9 kdsa
138.3 Tri
139.0 AA163_2
140.0 V16_1800
141.4 Clv2
142.7 AGL20a = soc1a
143.6 J14_1500
144.1 AC10_2
144.4 AE02_160
145.8 DHPS1
148.8 V17_1700
155.0 Aux1
161.8 RNAH
162.7 AB146
164.9 Viola1
165.6 ArfB3
171.2 AA399
176.2 Abi3
180.1 AC31_1
180.9 AE11_800
181.9 R04_950
182.5 A11_900
182.9 AD155
184.3 Gns2
185.5 AA176_3
186.0 AD55
186.8 AB142
187.1 AA255_2
188.2 AD175
192.3 AC58
194.5 COLa
197.7 mtGEN481
204.8 B20_300
206.2 Ls
208.8 AA460
215.9 Rbcs
0.0
1.5
4.7
4.9
19.9
26.2
27.1
31.2
36.8
41.1
41.5
43.9
51.8
54.4
56.3
58.7
63.1
64.4
71.4
72.5
74.0
74.9
76.8
78.8
79.5
81.7
84.1
87.6
89.3
90.8
94.3
96.1
98.6
100.2
100.3
101.2
104.2
104.5
105.3
106.0
107.2
108.0
111.2
114.1
119.6
124.1
127.6
132.0
134.3
137.1
139.3
142.0
154.9
157.3
160.2
161.0
162.5
163.3
164.4
166.0
167.3
171.4
181.6
182.5
185.5
199.0
V18_450
AB66-223 P16_2000
A14_680
UBC601B
AC10-185
cDNA187
AA228-328
AA81
AB23
U7_1500
U7_1700
AA189-125
Y15_200
D03_1100
AB47 (ns. *. ns)
V07_650
UBC601A
AC30-390
E01_870
O09_800 (ns. **. -)
AD02_950
AB40-715
S09_1650
AA255-750 (-. -. *)
AA99 (*. ns. -)
R03_2000
AD158
K02_750
AA475
UBC268B
AB130-219 (-. -. ***)
PSGAPA1
AD79 (ns. ***. ns)
AB83-360
AA163.2
V16_1800
UBC526
J14_1500 (ns. ***. -)
AE02_1600
UBC420
B11-465
B11-590
Y17_800
AD180-133
Y02_900 (-. *. -)
AD280-282
UBC352
AB146-346
UBC537
UBC603B
AA399
U7_1300 (-. -.*-)
AA98-660
R04_950
AD155-350
AB142-185 AD55.2
AD175
AA18-175
A11_900
AE11_800
AC58
Pgdc
B20_300
AA241-205
AA460 (*. ns. ns)
AA07_900

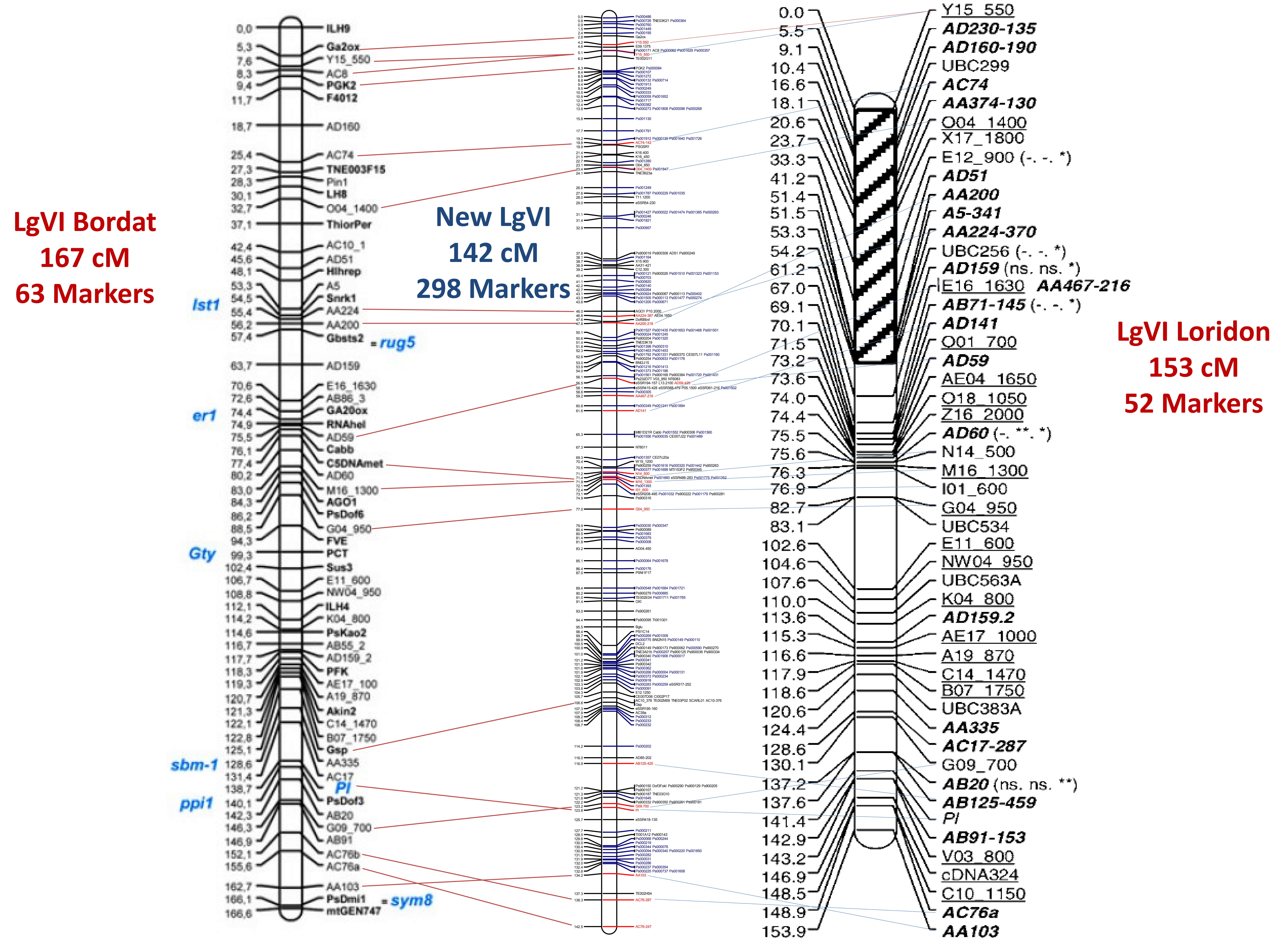

LgVI Bordat
167 cM
63 Markers
New LgVI
142 cM
298 Markers
LgVI Loridon
153 cM
52 Markers
Ist1
er1
Gty
sbm-1
ppi1
= rug5
= sym8
Pl
0,0 ILH9
5,3 Ga2ox
7,6 Y15_550
8,3 AC8
9,4 PGK2
11,7 F4012
18,7 AD160
25,4 AC74
27,3 TNE003F15
28,3 Pin1
30,1 LH8
32,7 O04_1400
37,1 ThiorPer
42,4 AC10_1
45,6 AD51
48,1 Hlhrep
53,3 A5
54,5 Snrk1
55,4 AA224
56,2 AA200
57,4 Gbsts2
63,7 AD159
70,6 E16_1630
72,6 AB86_3
74,4 GA20ox
74,9 RNAhel
75,5 AD59
76,1 Cabb
77,4 C5DNAmet
80,2 AD60
83,0 M16_1300
84,3 AGO1
86,2 PsDof6
88,5 G04_950
94,3 FVE
99,3 PCT
102,4 Sus3
106,7 E11_600
108,8 NW04_950
112,1 ILH4
114,2 K04_800
114,6 PsKao2
116,7 AB55_2
117,7 AD159_2
118,3 PFK
119,3 AE17_100
120,7 A19_870
121,3 Akin2
122,1 C14_1470
122,8 B07_1750
125,1 Gsp
128,6 AA335
131,4 AC17
138,7
140,1 PsDof3
142,3 AB20
146,3 G09_700
146,9 AB91
152,1 AC76b
155,6 AC76a
162,7 AA103
166,1 PsDmi1
166,6 mtGEN747
0.0 Y15_550
5.5 AD230-135
9.1 AD160-190
10.4 UBC299
16.6 AC74
18.1 AA374-130
20.6 O04_1400
23.7 X17_1800
33.3 E12_900 (-. -. *)
41.2 AD51
51.4 AA200
51.5 A5-341
53.3 AA224-370
54.2 UBC256 (-. -. *)
61.2 AD159 (ns. ns. *)
67.0 E16_1630 AA467-216
69.1 AB71-145 (-. -. *)
70.1 AD141
71.5 O01_700
73.2 AD59
73.6 AE04_1650
74.0 O18_1050
74.4 Z16_2000
75.5 AD60 (-. **. *)
75.6 N14_500
76.3 M16_1300
76.9 I01_600
82.7 G04_950
83.1 UBC534
102.6 E11_600
104.6 NW04_950
107.6 UBC563A
110.0 K04_800
113.6 AD159.2
115.3 AE17_1000
116.6 A19_870
117.9 C14_1470
118.6 B07_1750
120.6 UBC383A
124.4 AA335
128.6 AC17-287
130.1 G09_700
137.2 AB20 (ns. ns. **)
137.6 AB125-459
141.4 Pl
142.9 AB91-153
143.2 V03_800
146.9 cDNA324
148.5 C10_1150
148.9 AC76a
153.9 AA103

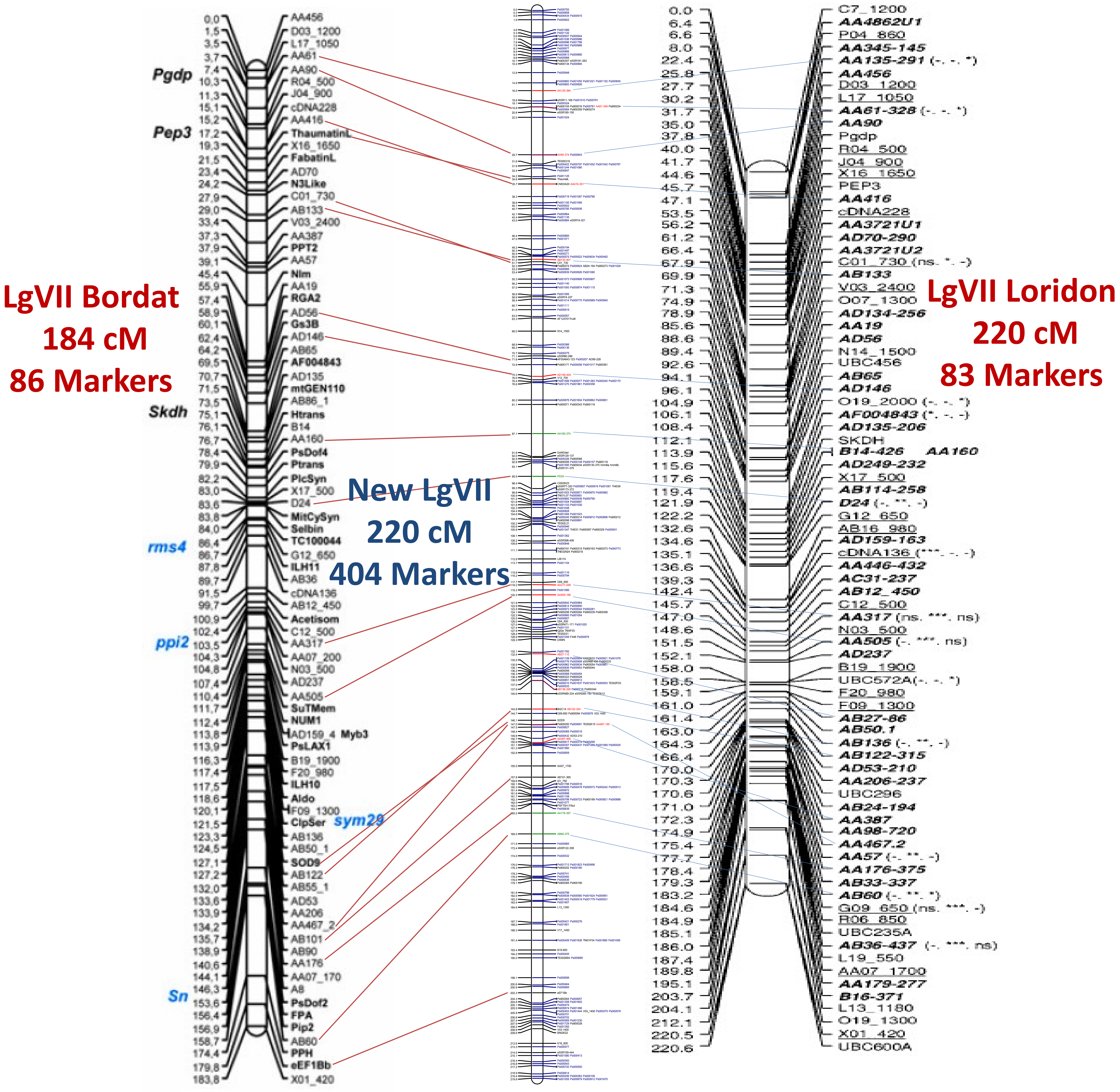
LgVII Bordat
184 cM
86 Markers
New LgVII
220 cM
404 Markers
LgVII Loridon
220 cM
83 Markers
Pgdp
Pep3
Skdh
rms4
ppi2
sym29
Sn
0,0 AA456
1,5 D03_1200
3,5 L17_1050
3,7 AA61
7,4 AA90
10,3 R04_500
11,3 J04_900
15,1 cDNA228
15,2 AA416
17,2 ThaumatinL
19,3 X16_1650
21,5 FabatinL
23,4 AD70
24,2 N3Like
27,9 C01_730
29,0 AB133
33,4 V03_2400
37,3 AA387
37,9 PPT2
39,1 AA57
45,4 Nlm
55,9 AA19
57,4 RGA2
58,9 AD56
60,1 Gs3B
62,4 AD146
64,2 AB65
69,5 AF004843
70,7 AD135
71,5 mtGEN110
73,5 AB86_1
75,1 Htrans
76,1 B14
76,7 AA160
78,4 PsDof4
79,9 Ptrans
82,2 PlcSyn
83,0 X17_500
83,6 D24
83,8 MitCySyn
84,0 Selbin
86,4 TC100044
86,7 G12_650
87,8 ILH11
89,7 AB36
91,5 cDNA136
99,7 AB12_450
100,9 Acetisom
102,4 C12_500
103,5 AA317
104,3 AA07_200
104,8 N03_500
107,4 AD237
110,4 AA505
111,7 SuTMem
112,4 NUM1
113,8 AD159_4 Myb3
113,9 PsLAX1
116,3 B19_1900
117,4 F20_980
117,5 ILH10
118,6 Aldo
120,1 F09_1300
121,5 ClpSer
123,3 AB136
124,5 AB50_1
127,1 SOD9
127,2 AB122
132,0 AB55_1
133,6 AD53
133,9 AA206
134,2 AA467_2
135,7 AB101
138,9 AB90
140,6 AA176
144,1 AA07_170
146,3 A8
153,6 PsDof2
156,4 FPA
156,9 Pip2
158,7 AB60
174,4 PPH
179,8 eEF1Bb
183,8 X01_420
0.0 C7_1200
6.4 AA4862U1
6.6 P04_860
8.0 AA345-145
22.4 AA135-291 (-, -, *)
25.8 AA456
27.7 D03_1200
30.2 L17_1050
31.7 AA61-328 (-, -, *)
35.0 AA90
37.8 Pgdp
40.0 R04_500
41.7 J04_900
44.6 X16_1650
45.7 PEP3
47.1 AA416
53.5 cDNA228
56.2 AA3721U1
61.2 AD70-290
66.4 AA3721U2
67.9 C01_730 (ns, *, -)
69.9 AB133
71.3 V03_2400
74.9 O07_1300
78.9 AD134-256
85.6 AA19
88.6 AD56
89.4 N14_1500
92.6 UBC456
94.1 AB65
96.1 AD146
104.9 O19_2000 (-, -, *)
106.1 AF004843 (*, -, -)
108.4 AD135-206
112.1 SKDH
113.9 B14-426 AA160
115.6 AD249-232
117.6 X17_500
119.4 AB114-258
121.9 D24 (-, **, -)
122.2 G12_650
132.6 AB16_980
134.6 AD159-163
135.1 cDNA136 (***, -, -)
136.6 AA446-432
139.3 AC31-237
142.4 AB12_450
145.7 C12_500
147.0 AA317 (ns, ***, ns)
148.6 N03_500
151.5 AA505 (-, ***, ns)
152.1 AD237
158.0 B19_1900
158.5 UBC572A(-, -, *)
159.1 F20_980
161.0 F09_1300
161.4 AB27-86
163.0 AB50.1
164.3 AB136 (-, **, -)
166.4 AB122-315
170.0 AD53-210
170.3 AA206-237
170.6 UBC296
171.0 AB24-194
172.3 AA387
174.9 AA98-720
175.4 AA467.2
177.7 AA57 (-, **, -)
178.4 AA176-375
179.3 AB33-337
183.2 AB60 (-, **, *)
184.6 G09_650 (ns, ***, -)
184.9 R06_850
185.1 UBC235A
186.0 AB36-437 (-, ***, ns)
187.4 L19_550
189.8 AA07_1700
195.1 AA179-277
203.7 B16-371
204.1 L13_1180
212.1 O19_1300
220.5 X01_420
220.6 UBC600A
