# Supplementary material for: Transcriptome sequencing for high throughput SNP development and genetic mapping in Pea
Source: BMC Genomics. 2014 Feb 12;15:126. doi: 10.1186/1471-2164-15-126 (PMC3925251; doi:10.1186/1471-2164-15-126)
Supplement: Additional file 14: Figure S8 — Colinearity of common markers between our study (middle) and Bordat et al. ([39]; left) and Loridon et al. ([38]; right) composite maps. [file 1471-2164-15-126-S14.pdf]

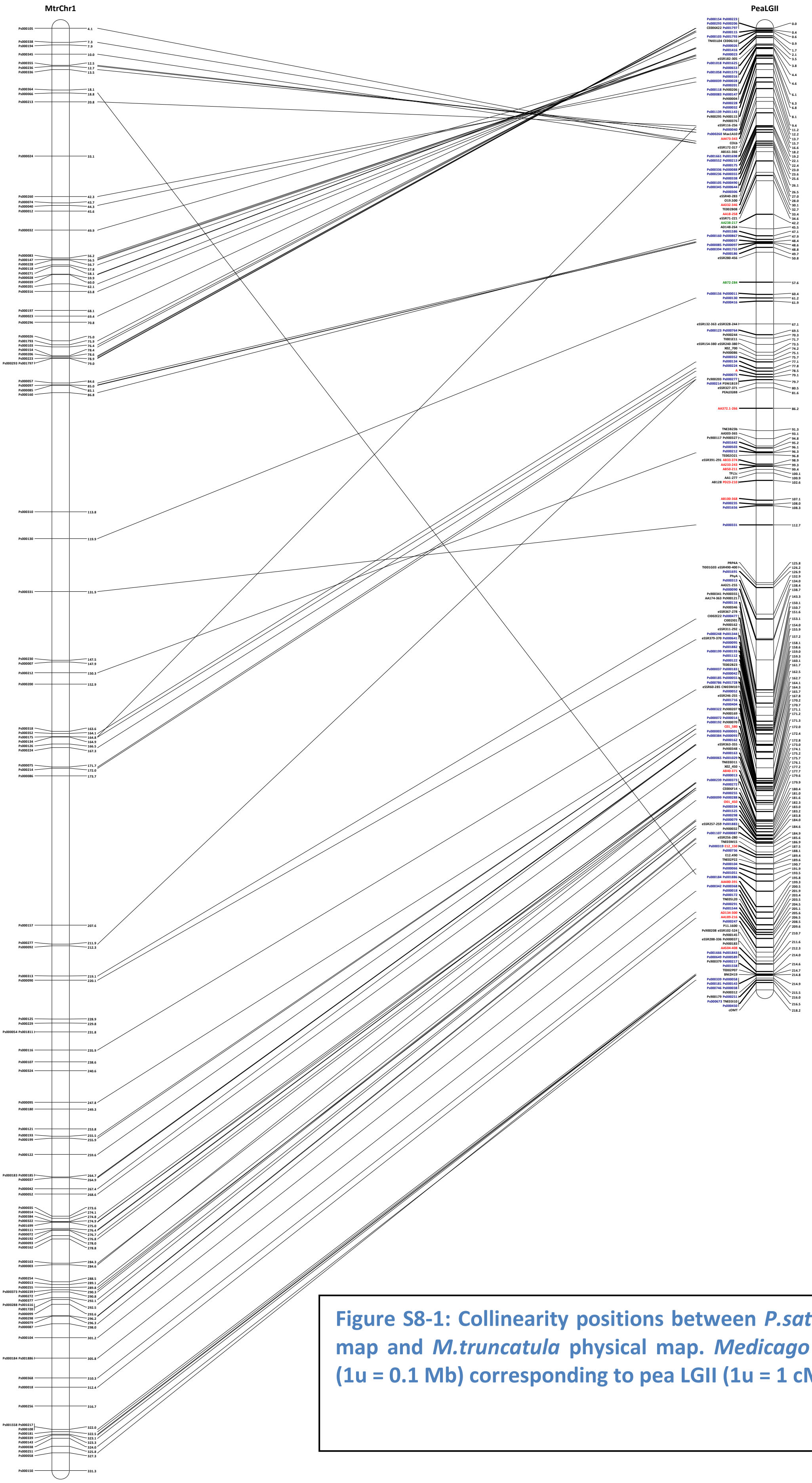

Figure S8-1: Collinearity positions between *P.sativum* composite genetic map and *M.truncatula* physical map. *Medicago* pseudochormrosome 1 (1u = 0.1 Mb) corresponding to pea LGII (1u = 1 cM)

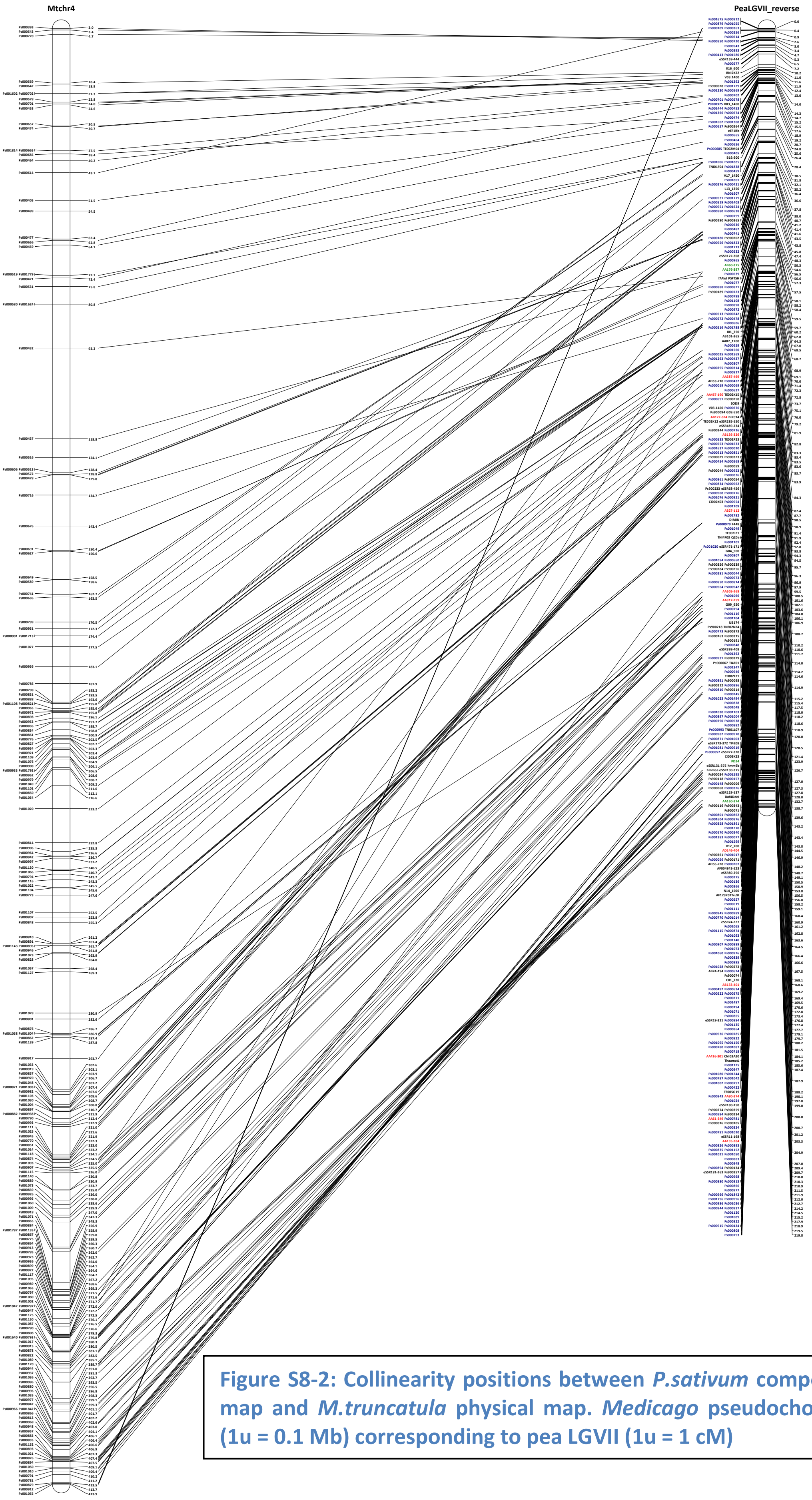

Figure S8-2: Collinearity positions between *P.sativum* composite genetic map and *M.truncatula* physical map. *Medicago* pseudochormrosome 4 (1u = 0.1 Mb) corresponding to pea LGVII (1u = 1 cM)

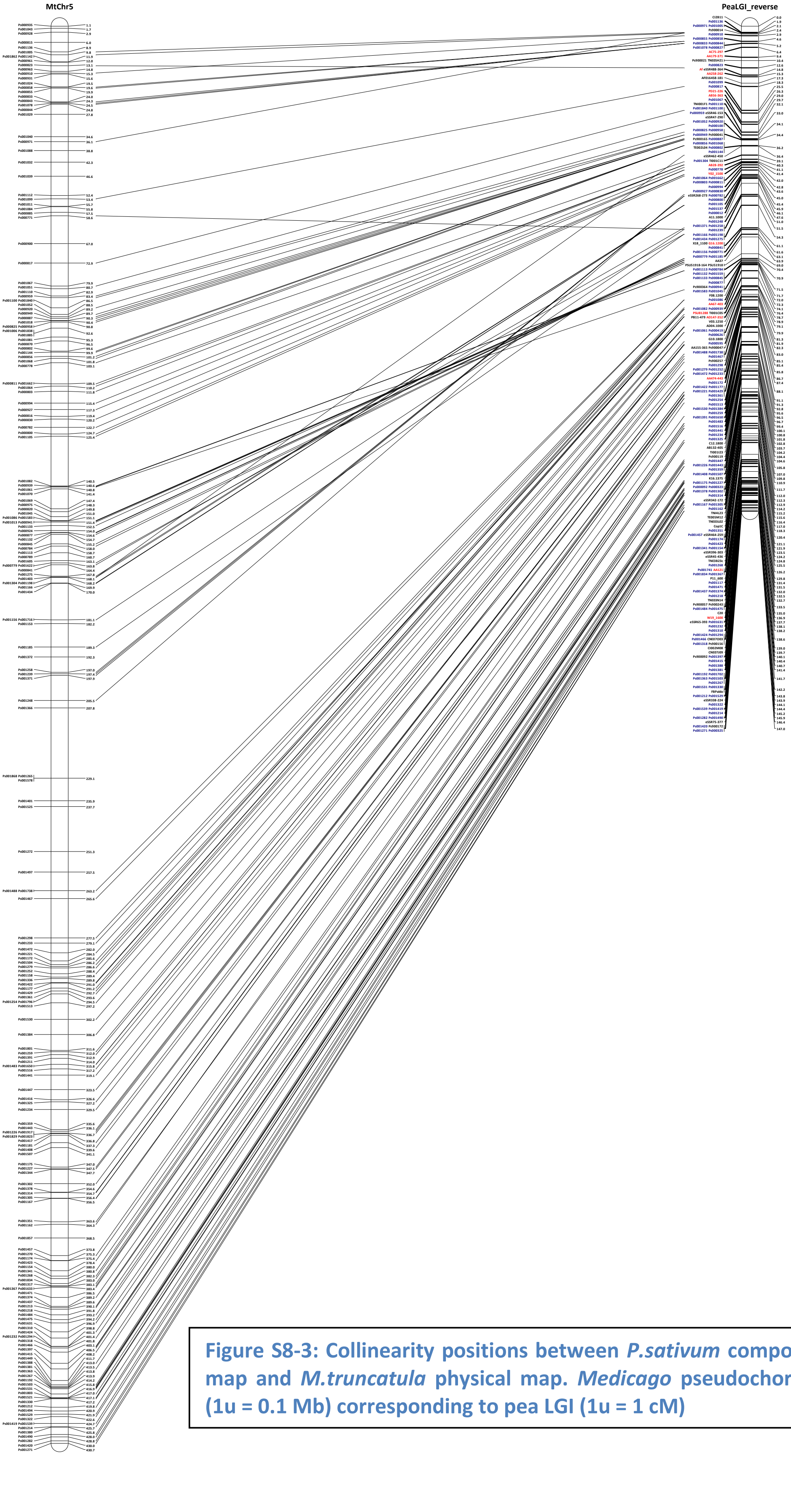

Figure S8-3: Collinearity positions between *P.sativum* composite genetic map and *M.truncatula* physical map. *Medicago* pseudochormrosome 5 (1u = 0.1 Mb) corresponding to pea LGI (1u = 1 cM)

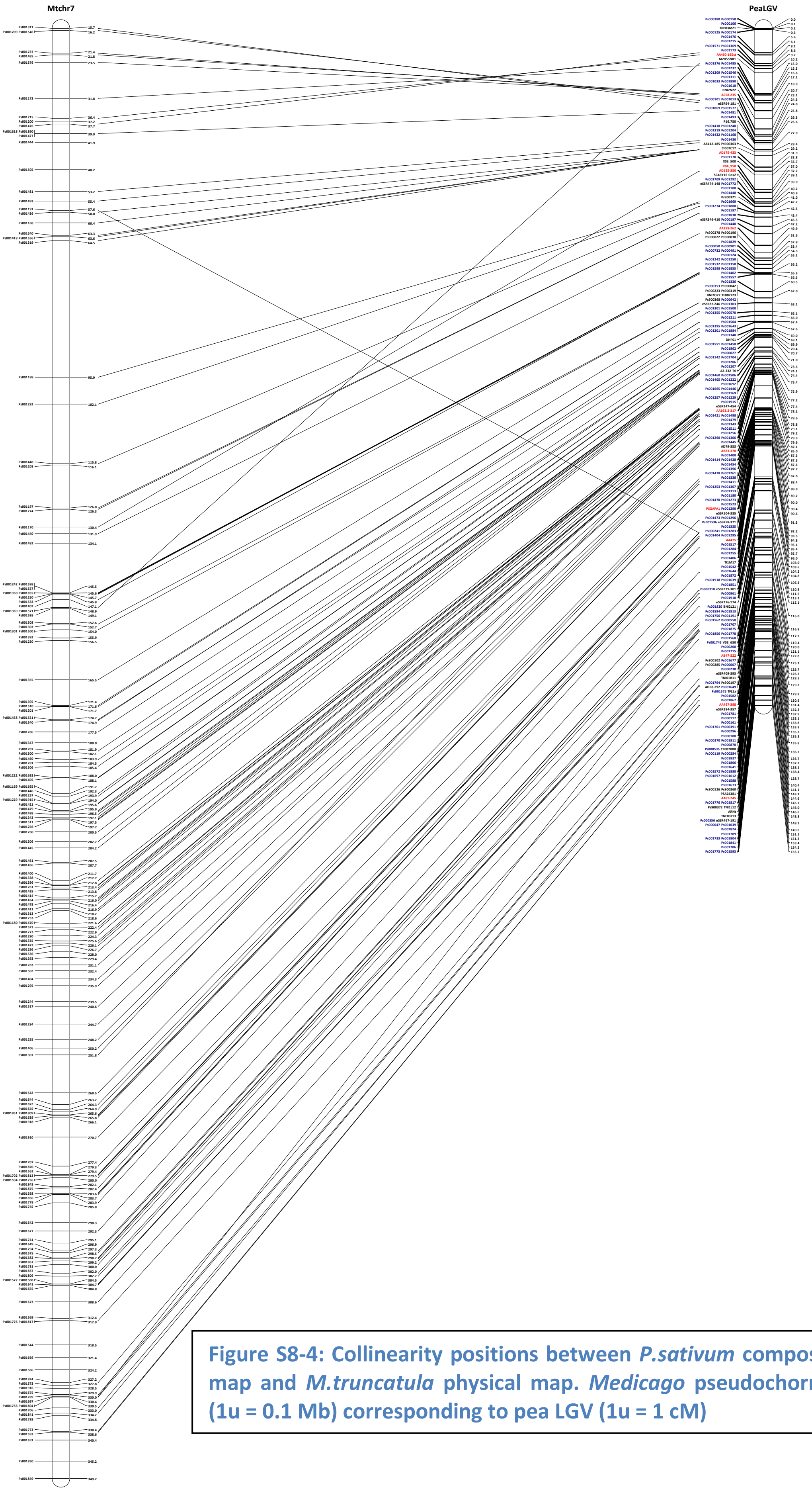

Figure S8-4: Collinearity positions between *P.sativum* composite genetic map and *M.truncatula* physical map. *Medicago* pseudochormrosome 7 (1u = 0.1 Mb) corresponding to pea LGV (1u = 1 cM)

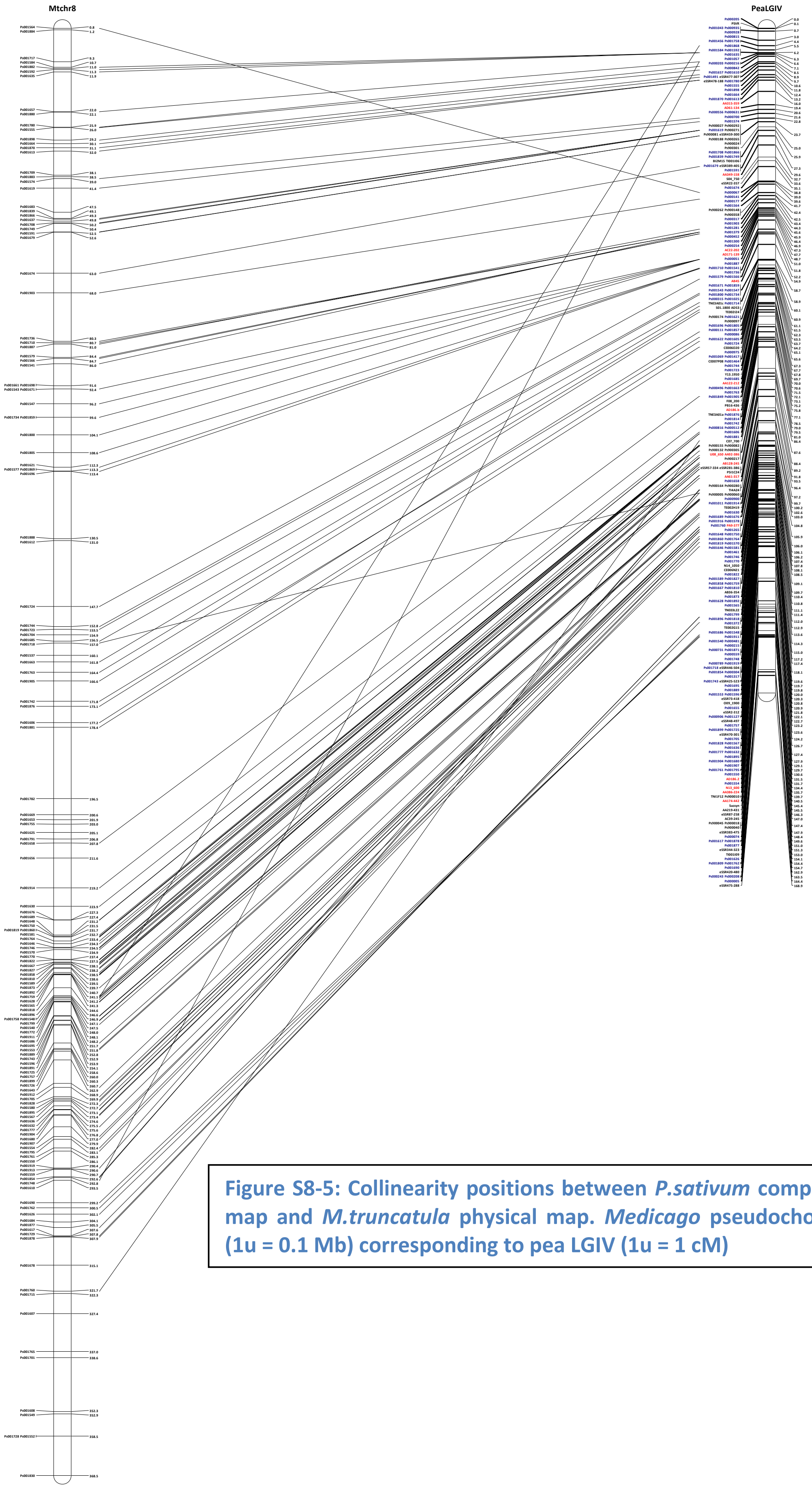

Figure S8-5: Collinearity positions between *P.sativum* composite genetic map and *M.truncatula* physical map. *Medicago* pseudochormrosome 8 (1u = 0.1 Mb) corresponding to pea LGIV (1u = 1 cM)
