# Supplementary material for: Transcriptome sequencing for high throughput SNP development and genetic mapping in Pea
Source: BMC Genomics. 2014 Feb 12;15:126. doi: 10.1186/1471-2164-15-126 (PMC3925251; doi:10.1186/1471-2164-15-126)
Supplement: Additional file 17: Table S6 — List of Accessions used for sequencing and/or genotyping. [file 1471-2164-15-126-S17.pdf]

	Name	Synonyme	Country origin	Breeding comp	Reg date (1)	Status (2)	End Use (3)	Sowing type (4)	Foliage (5)	Flower col (6)	Cot col (7)	Photop (8)
C1-1	NETTE		Belgium	Limagrain	2008	CV	Dry	Sp	af	W	Y	
C1-1	TUDOR		Netherlands	Limagrain	2003	CV	Dry	Sp	af	W	Y	
C1-1	RESPECT		France	Danisco / AO	2006-Aut	CV	Dry	Sp	af	W	Y	
C1-1	BLUEMOON		France	Danisco / AO	2007-GB	CV	Dry	Sp	af	W	G	
C1-1	SALAMANCA		France	Nord. Pflanz.	2010	CV	Dry	Sp	af	W	Y	
C1-1	MYTHIC		France	Toft PB	2010	CV	Dry	Sp	af	W	Y	
C1-1	MANTARA		Netherlands	Limagrain	2008	CV	Pigeon	Sp	af	W	Y	
C1-1	AVENUE		France	Nord. Pflanz.	2010	CV	Dry	Sp	af	W	Y	
C1-1	GARDE		Netherlands	Limagrain	2001	CV	Dry	Sp	af	W	G	
C1-1	ALEZAN		France	Unisigma	2003	CV	Dry	Sp	af	W	Y	
C1-1	SOLIDO		Netherlands	Limagrain	1993	CV	marrowfat	Sp	af	W	G	
C1-1	INDIANA	S04 H088	France	Serasem	2010	CV	Dry	W	af	W	Y	hr
C1-1	STRADA		Netherlands	Limagrain	2010	CV	marrowfat	Sp	af	W	G	
C1-1	NEON		Netherlands	Limagrain	2010	CV	marrowfat	Sp	af	W	G	
C1-1	SCHOLAR	LAN2033	Netherlands	Limagrain	2012	Bl	marrowfat	Sp	af	W	G	
C1-1	TERESE			Pajbjergfonden	1988	CV	Dry	Sp	af	W		
C1-1	MONTANA		France	Cebeco	1990	CV	Dry	Sp	af	W	Y	
C1-1	CARRERRA		France	Cebeco	1992	CV	Dry	Sp	af	W	Y	
C1-1	AUSTIN		France	Nickerson	1998	CV	Dry	Sp	af	W	Y	
C1-1	BADMINTON		France	Desprez	1995	CV	Dry	Sp	af	W	Y	
C1-1	ANTARA	LN4211	Netherlands	Limagrain	2013	CV	Dry	Sp	af	W	Y	
C1-1	PROPHET		Netherlands	Limagrain	2005	CV	Dry	Sp	af	W	G	
C1-1	SPIDER		Netherlands	Limagrain	2005	CV	Dry	Sp	af	W	Y	
C1-1	CRACKERJACK		France	Danisco / AO	2007-GB	CV	Dry	Sp	af	W	G	
C1-1	FUSION		Netherlands	Limagrain	2006	CV	Dry	Sp	af	W	Y	
C1-1	ECLIPSE		France	Cebeco	2001	CV	Dry	Sp	af	W	Y	
C1-1	MATRIX		Netherlands	Limagrain	2004?	CV	Dry	Sp	af	W	G	
C1-1	AUDIT		France	Nickerson	2009	CV	Dry	Sp	af	W	Y	
C1-2	QUADRIL		France	lovis Matton/Genseec	2010	CV	Dry	Sp	af	W	Y	
C1-2	BLUESTAR		France	Serasem	2008-GB	CV	Dry	Sp	af	W	G	
C1-2	ARDAN		France	Unisigma	2011	CV	Dry	Sp		W	Y	
C1-2	HARDY		France	Serasem	2001	CV	Dry	Sp	af	W	Y	
C1-2	BOLDOR		France	Desprez	2010	CV	Dry	Sp	af	W	Y	
C1-2	KENZZO		France	Momont/Actisem	2010	CV	Dry	Sp	af	W	Y	
C1-2	STARTER		France	Serasem	2006	CV	Dry	Sp	af	W	Y	
C1-2	REBEL		Belgium	Limagrain	2011	CV	Dry	Sp	af	W	Y	
C1-2	ABARTH	LN4206	Netherlands	Limagrain	2011	CV	Dry	Sp	af	W	Y	
C1-2	COOPER		Netherlands	Innoseeds	2003	CV	Dry	Sp	af	W	G	
C1-2	KAYANNE		France	L.Petkus/Momont	2008	CV	Dry	Sp	af	W	Y	
C1-2	AVANTGARDE		France	Nickerson	2010	CV	Dry	Sp	af	W	Y	
C1-2	SATELIT		France	lovis Matton/Seraser	2010	CV	Dry	Sp	af	W	Y	
C1-2	AUCKLAND		Netherlands	Limagrain	2009	CV	Dry	sp	af	W	Y	
C1-2	EDEN		France	Danisco / Nickerson	2007	CV	Dry	Sp	af	W	Y	
C1-2	BRIDGE		France	Desprez	1992	CV	Dry	Sp	af	W	Y	
C1-2	ATHOS		France	Nickerson	1997	CV	Dry	Sp	af	W	Y	
C1-2	BILBOQUET		France	Desprez	2002	CV	Dry	Sp	af	W	Y	
C1-2	BACCARA		France	Desprez	1991	CV	Dry	Sp	af	W	Y	
C1-2	JAVLO		France	Desprez	1999	CV	Dry	Sp	af	W	Y	
C1-2	MARIGNAN		France	Unisigma	2007	CV	Dry	Sp	af	W	Y	
C1-2	LUMINA		France	Nickerson	2001	CV	Dry	Sp	af	W	Y	
C1-2	POLSTEAD		Netherlands	Limagrain	2004	CV	Dry	Sp	af	W	Y	
C1-2	NAVARRO		France	Serasem	2010	CV	Dry	Sp	af	W	Y	
C1-2	GREGOR		France	Serasem	2007	CV	Dry	Sp	af	W	Y	
C1-2	ONYX		France	Serasem	2008	CV	Dry	Sp	af	W	Y	
C1-2	MOWGLI	SR4268-3		Serasem		Bl				W		
C1-3	COMANCHE	SR06H086	France	Serasem	2011	CV	Dry	W	af	W	Y	hr
C1-3	ASTRONAUTE	SR5149-9		Serasem		Bl	Dry	Sp	af	W		
C2-1	AeD99OSW-45-8-7			GSP		Bl	Dry	Sp				
C2-1	552	GSP1070	USA			Bl	Gd	Sp				
C2-1	AeD99OSW-50-2-5			GSP		Bl	Dry	Sp				
C2-1	PUGET					CV	Gd	Sp	Af	W		
C2-1	DSP	9411-340-B				CV	Gd	Sp	Af	W		
C2-1	90-2079		USA	USDA		Bl	Gd	Sp	Af	W		
C2-1	831-08					Bl						
C2-1	PI180693					Gmp		Sp	Af	P		
C2-1	90-2131		USA	USDA		Bl	Gd	Sp		W		
C2-1	LISA					Gmp						
C2-1	AeD99QU-04-8v-2-5			GSP		Bl	Dry	Sp				
C2-1	CHINA					Gmp	Gd	W	Af			
C2-1	E11					Gmp						
C2-1	JI296		France			CV	Gd	Sp	Af	W		
C2-1	CAMEOR					CV	Gd	Sp	Af	W		
C2-2	FD04505-03		France	Desprez		Bl	Dry	W	af	W		hr
C2-2	ISARD		France	AO	2005	CV	Dry	W	af	W	Y	hr
C2-2	GANGSTER	RLH07070	France	RAGT	2012	CV	Dry	W	af	W	Y	hr
C2-2	JAMES		France	Serasem	2008	CV	Dry	W	af	W		hr
C2-2	GERONIMO	S04 H207	France	Serasem	2010	CV	Dry	W	af	W	G	Hr
C2-2	SPENCER	RLH09331	France	RAGT	2012	CV	Dry	W	af	W	Y	Hr
C2-2	DP					Gmp	Fd	W	Af	P		Hr
C2-2	CHAMPAGNE					CV	Fd	W	Af	P		Hr
C2-2	886/01		France	AO		Bl	Dry	W		W		Hr
C2-2	CHEROKEE		France	GAE	2005	CV	Dry	W	af	W	Y	hr
C2-2	CURLING	FD20	France	Desprez	2012	CV	Dry	W	af	W	Y	hr
C2-2	AVIRON	FD03	France	Desprez	2012	CV	Dry	W	af	W	G	hr
C2-2	ENDURO		France	Desprez	2006	CV	Dry	W	af	W	Y	hr
C2-2	LUCY		France	GAE	2000	CV	Dry	W	af	W	G	hr

(1) Registration date; (2) Cultivated status: Cv = cultivar, Bl = Breeding Line, Lv = local variety, Gmp = Germplasm; (3) End-use type: Fd = Fodder, Gd = Garden; (4) Sowing type: W = winter sown, Sp = spring sown; (5) Foliage: Nl = normal leaf, af = afila, rg = rogue; (6) Flower colour: P=purple, W=white ; (7) Cotyledon colour: G = green, Y = yellow ; (8) Photoperiod : Hr : highly responsive, hr = not responsive
